# Arabidopsis RAD16 Homologues Are Involved in UV Tolerance and Growth

**DOI:** 10.3390/genes14081552

**Published:** 2023-07-28

**Authors:** Linda Alrayes, Jake Stout, Dana Schroeder

**Affiliations:** Department of Biological Sciences, University of Manitoba, Winnipeg, MB R3T 2N2, Canada; jake.stout@umanitoba.ca (J.S.); dana.schroeder@umanitoba.ca (D.S.)

**Keywords:** RAD7/16, DDB1/2, CSA/CSB, *Arabidopsis*, Nucleotide excision repair, UV sensitivity

## Abstract

In plants, prolonged exposure to ultraviolet (UV) radiation causes harmful DNA lesions. Nucleotide excision repair (NER) is an important DNA repair mechanism that operates via two pathways: transcription coupled repair (TC-NER) and global genomic repair (GG-NER). In plants and mammals, TC-NER is initiated by the Cockayne Syndrome A and B (CSA/CSB) complex, whereas GG-NER is initiated by the Damaged DNA Binding protein 1/2 (DDB1/2) complex. In the yeast *Saccharomyces cerevisiae* (*S. cerevisiae*), GG-NER is initiated by the Radiation Sensitive 7 and 16, (RAD7/16) complex. *Arabidopsis thaliana* has two homologues of yeast RAD16, At1g05120 and At1g02670, which we named AtRAD16 and AtRAD16b, respectively. In this study, we characterized the roles of AtRAD16 and AtRAD16b. *Arabidopsis rad16* and *rad16b* null mutants exhibited increased UV sensitivity. Moreover, AtRAD16 overexpression increased plant UV tolerance. Thus, AtRAD16 and AtRAD16b contribute to plant UV tolerance and growth. Additionally, we found physical interaction between AtRAD16 and AtRAD7. Thus, the *Arabidopsis* RAD7/16 complex is functional in plant NER. Furthermore, AtRAD16 makes a significant contribution to *Arabidopsis* UV tolerance compared to the DDB1/2 and the CSB pathways. This is the first time the role and interaction of DDB1/2, RAD7/16, and CSA/CSB components in a single system have been studied.

## 1. Introduction

Plants are sessile organisms that must adapt and respond to their environment to successfully survive. Sunlight is an important part of a plant’s environment; it contains both useful visible light that plants use for growth and development and harmful ultraviolet (UV) light. UV light has pleiotropic effects on plant development and can damage cellular structures including DNA. UV-induced DNA damage can alter transcription, replication, and cause genome instability [1].

Plants utilize two different repair mechanisms to repair UV-damaged DNA and maintain genome integrity: light repair via photolyases [2], and dark repair via NER. NER is a five-step process consisting of initial recognition of UV-damaged DNA, helicase activity to open the double helix, excision of the damaged base, incorporation of the correct base-paired nucleotide, and the final ligation of the repaired strand. NER is a well conserved repair process across both prokaryotes and eukaryotes. Defects in NER and failure to remove lesions from the genome cause xeroderma pigmentosa (UV-sensitivity with increased skin cancer risk) in humans [3], UV hypersensitivity (*uvh*) in *Arabidopsis thaliana* [4], and radiation sensitivity (*rad*) in the yeast *Saccharomyces cerevisiae* [5].

NER utilizes two distinct sub-pathways: transcription coupled repair (TC-NER) and global genomic repair (GG-NER), which are responsible for the repair of transcriptionally active areas and transcriptionally silent areas, respectively [5,6,7]. In mammalian TC-NER, the Cockayne Syndrome A and B proteins (CSA and CSB), as well as the UV-stimulated scaffold protein A (UVSSA), are required to displace the stalled RNA polymerase from the damaged site, to allow NER proteins access to the lesion to proceed with the repair [8,9]. In yeast, TC-NER is initiated by RAD28 and RAD26, the yeast homologues of mammalian CSA and CSB [10]. The downstream repair components are also well conserved between mammals and yeast [11].

In mammals, the damage recognition complex that initiates GG-NER is comprised of DDB1, DDB2, Cullin4 (CUL4), and RING-BOX1 (RBX1) [7,12]. This complex has E3 ubiquitin ligase activity that targets core histone proteins for chromatin modification, resulting in the recruitment and ubiquitination of Xeroderma Pigmentosa C (XPC) [13,14]. Then XPC, together with HR23B and Centrin2 (CEN2) proteins, form another complex that recruits the NER machinery [4,15]. *S*. *cerevisiae* has homologues of human XPC and HR23B, named ScRAD4 and ScRAD23, respectively, and their roles in the NER pathway are well conserved [5]. However, no homologues of the initial damage recognition complex DDB1, DDB2, and CUL4 exist in yeast [16]. Instead, another complex consisting of Radiation Sensitive 7 (RAD7), RAD16, Elongin c (ELC1), and Cullin3 (CUL3) plays similar recognition roles in yeast, such as chromatin modification and RAD4 recruitment [17]. Although RAD7 and RAD16 have the same function as DDB1 and DDB2, these proteins are not direct homologs (respectively).

Nucleotide excision repair in plants has been studied in *Arabidopsis thaliana*, and the overall process appears to be well conserved [4]. Current understanding of this process is that it follows the mammalian model of GG-NER, given that mutations in the homologs of the core downstream NER components CEN2 and RAD23 result in UV sensitivity [18,19]. Loss of function mutations in genes encoding the components of DNA damage recognition (*DDB1*, *DDB2*, *CUL4*) also results in UV sensitivity [20,21,22,23]. However, the *Arabidopsis* genome also contains homologs of the yeast DNA damage recognition machinery: three homologs of RAD7, two homologs of RAD16, two homologs of CUL3, and one Elongin C homologue [24,25,26]. The presence of RAD16, RAD7, ELC, and DDB1/2 homologues in the *Arabidopsis* genome provide a unique opportunity to examine the role and interaction of both the mammalian homologs DDB1/2 and yeast homologs (RAD7/16) in DNA damage recognition in a single system.

Thus, the goal of this study is to characterize the roles of the *Arabidopsis* RAD16 homologues in UV tolerance and growth, to address the potential redundancy between *Arabidopsis RAD16* homlogs, and to investigate the relative contribution of AtRAD16 to plant UV tolerance compared to the AtDDB1/2 and AtCSB repair pathways. Finally, to detect physical interactions within Arabidopsis RAD7/16 complex and between AtRAD16 and other NER proteins.

## 2. Materials and Methods 

### 2.1. Sequence Analysis 

Amino acid sequences for RAD16 homologs were downloaded from public databases. The Pfam database online was used to obtain the protein domains. Publicly available expression databases were obtained from http://bar.utoronto.ca/interactions/cgibin/Arabidopsis_interactions_viewer.cgi [Accessed 20 April 2020] to determine tissue-specific and stress-responsive expression in *Arabidopsis*. 

### 2.2. Plant Materials and Growth Conditions

All T-DNA insertion alleles and the wild type control (*Col*-*0*) used in this study were ordered from the Arabidopsis Biological Resource Center (ABRC): *rad16*-*2* (SALK_130522.34.85x in At1g05120), *rad16b*-*1* (SALK_147762 in At1g02670), *rad7a*-*3* (SALK_107725 in At2g06040), *rad7c*-*1* (SALK_025534 in At5g21900), *ddb2*-*1* (SALK_040408 in At5g58760), *uvssa*-*2* (SALK_061538 in At3g61800), and *csb*-*1* (SALK_000799 in At2g18760), *rad23b*-*1* (SALK_076360 in At1g79650).

All insertion mutant lines were genotyped using allele-specific primers, along with the T-DNA-specific primer LBb1.3 (Table S1). Seeds were surface sterilized and spread on Linsmaier and Skoog (LS) media with 0.86% phytoblend and 0.6% sucrose. Following stratification at 4 °C for two days, the plates were transferred to 20 °C under fluorescent lights (100 μM photons m^−2^ s^−1^) at 50% relative humidity. Seedlings were transplanted after 14 days into Sunshine mix #1 (SunGro, Bellevue, WA, USA). Both seedlings and adults were grown under long day conditions (16 h light/8 h dark).

### 2.3. RNA Extraction and RT-PCR 

Total RNA from 50 seven-day-old whole seedlings/genotype were extracted using the E.Z.N.A. Plant RNA Kit (Omega Bio-Tek, GA, USA), according to manufacturer instructions, which included a DNase treatment. The Maxima First Strand cDNA synthesis kit (Fermentas, Waltham, MA, USA) was used to generate cDNA. For semi-quantitative RT-PCR, *RAD16* and *RAD16b* transcript abundance was estimated by PCR amplification for 25 cycles using gene-specific primers (Table S1). The loading control actin was used, and all reactions were amplified for 24 cycles using the primers listed in Table S1. SsoFast EvaGreen Supermix (Bio-Rad, Hercules, CA, USA) was used to perform RT-PCR using *RAD16* cDNA specific primers listed in Table S1. The CFX Connect Real-time PCR detection system (Bio-Rad, Hercules, CA, USA) was used for analysis. The EF1α gene (At5g60390) was used as a control to normalize the RT-qPCR data [27,28]. The mean values were calculated using three biological replicates; each was performed in triplicate and the technical replicates were calculated.

### 2.4. UV Sensitivity Assays

For hypocotyl and root UV sensitivity assays, seeds were surface sterilized and spread on LS media with 0.86% phytoblend and 0.6% sucrose, stratified for two days at 4 °C, and grown vertically for four days at 20 °C, before being irradiated with varying doses of UV-C emitted by a shortwave UV lamp (XX-15S, UVP/LLC, Upland, CA, USA). After that, to avoid photoreactivation, irradiated plates were wrapped in aluminum foil, then rotated 90° and incubated vertically on either light or dark conditions for the indicated period. Plates were scanned and new root growth and hypocotyl length were measured using ImageJ (1.36b NIH, USA).

For adult plants UV sensitivity assays, 14 day-old-seedlings were transferred to soil. After 21 days of growth, the plants were irradiated with either 0, 300, 500, or 600 J m^−2^ UV-C. After incubation in the dark for three days, the plants were then returned to standard growth conditions (16 h light/8 h dark). After four days of growth under standard growth conditions, leaf damage was scored. Any leaf exhibiting brown or yellow coloration was scored as a damaged leaf, and the percent of undamaged leaves (undamaged leaves—green)/undamaged and damaged leaves—green and brown or yellow) was computed for twelve plants of each genotype and each treatment.

### 2.5. Adult Growth and Developmental Parameters 

Seedlings were grown for two weeks on LS media then transferred to Sunshine Mix Number 1 soil (SunGro, Bellevue, WA, USA), and 12 plants per line were used to collect the following data: flowering time-days (number of days it took for buds to appear), flowering time-leaves (the number of rosette and cauline leaves at flowering), rosette diameter was measured at 28 days of growth. Apical dominance (number of stems), plant height, and silique length were measured at seven weeks of growth.

### 2.6. Generation of RAD16 Overexpression Lines

A *RAD16* cDNA in pCMV SPORT6.1 vector was obtained from the French Institut National de la Recherche Agronomie. The cDNA was PCR-amplified with primers that added Sfil inkers and cloned into the pENTR223.1-Sfi vector. Then, LR recombinase was used to clone the *RAD16* cDNA into pEarleyGate100 (CaMV 35S promoter) and pEarleyGate104 (CaMV 35S promoter with an N-terminal yellow florescent protein (YFP) tag [29]. The overexpression vectors were then transformed into *Agrobacterium tumefaciens* GV3101strain, and flowering wild type (*Col*-*0*) and *Atrad16*-*2* were sprayed with *Agrobacterium* [30]. Basta selection was used to screen the transformed T1, as well as to screen for T2 lines carrying a single-copy transgene insertion. Homozygous T3 plants were used for subsequent experiments. 

### 2.7. Protein Subcellular Localization

YFP-tagged RAD16 seeds were plated on LS media, incubated at 4 °C for two days, then exposed to light for 6 h at 20 °C. Following light treatment, plates were wrapped in two layers of foil and returned to the incubator for three days. Three seedlings were placed on a slide; a drop of distilled water was added, observed using a Zeiss AXIO Imager Z1 Microscope (Zeiss, Oberkochen, Germany) equipped with AxioVision 4.8 software, using YFP (Filter Set YFP-2427B-000), and DAPI (Zeiss Filter Set 02 (488002-9901-000) filters (Semrock Inc., Rochester, NY, USA). Hoechst 33342 staining was used to visualize the nucleus [31]. 5 µg/mL Hoechst staining was prepared, and seedlings were immersed in the prepared staining for 30 min and imaged. For UV treatment, seedlings were treated with 1000 J m^−2^ UV-C, some were incubated in the dark for one hour and some for two hours then observed.

### 2.8. Yeast Two-Hybrid Screening

The GAL-4 Matchmaker gold yeast two-hybrid system (Clontech, Mountian View, CA, USA) was used to investigate *Arabidopsis* RAD16 protein interactions with other *Arabidopsis* GG-NER components (RA7a, RAD7b, RAD7c, RAD4, RAD23b, ELOC, and DDB2). *Arabidopsis RAD16*, and *RAD7a* cDNAs were PCR-amplified with primers that added Sfil inkers and cloned into the pENTR223.1-Sfi entry vector using T4 ligase. Subsequent clones were produced using LR recombinase to clone each gene cloned into pGADT7-DEST (Leu selection) prey vector and pGBKT7-DEST (Trp selection) bait vector [32]. These clones were then transformed into haploid yeast strains Y2HGold and Y187 using electroporation. The pGBKT7-DEST and pGADT7-DEST vectors were kindly provided by Yuhai Cui (Agriculture and Agri-Food Canada, London, ON). RAD7b, RAD7c, RAD4, RAD23b, ELOC, and DDB2 expressing strains were previously prepared by other students in our lab. Haploid yeast cells were mated to generate diploid strains as follows; *AtRAD16* pGADT7 was mated with *AtRAD7a* pGBKT7, *AtRAD7b* pGBKT7, *AtRAD4* pGBKT7, *AtRAD7c* pGBKT7, *AtDDB2* pGBKT7, *AtELOC* pGBKT7, and *AtRAD23b* pGBKT7. *AtRAD16* pGBKT7 was mated with *AtRAD7a* pGADT7, *AtRAD7b* pGADT7, *AtRAD7c* pGADT7, *AtRAD4* pGADT7, *AtDDB2* pGADT7, *AtELOC* pGADT7, and *AtRAD23b* pGADT7.

Interaction between proteins in diploid yeast were detected by selecting them on a quadruple drop-out medium (–ade, –his, –leu, –trp), and double drop-out (-trp -leu) medium was used as a control. p53/T (murine p53/SV40 large T-antigen) and Lam/T (Lamin/SV40 large T-antigen) were used as the positive and negative controls, respectively. Protein/protein interaction induces the expression of *ADE2* and *HIS3* reporters, which allows for growth on quadruple drop-out selection medium (-ade –his –leu -trp).

### 2.9. Generation of Double Mutant Plants

Double mutant plants were generated by crossing their corresponding homozygous single parental mutants to generate F1 heterozygotes. After selfing, the F2 generation was genotyped to identify double loss of function mutants. Homozygous F3 plants were used for subsequent experiments.

### 2.10. Statistical Analysis

All analyses were performed using a two-tailed Student’s *t*-test in Microsoft Excel. Probabilities of 0.05 or less were used to assess statistical significance. All experiments were repeated at least twice.

## 3. Results

### 3.1. Arabidopsis RAD16 Proteins Are Homologous to Yeast RAD16 Proteins

Two homologues of yeast RAD16 exist in *Arabidopsis thaliana* encoded by At1g05120 and At1g02670 [25]. An amino acid sequence alignment with *S*. *cerevisiae* RAD16 and *S*. *pombe* 16 homologue RHP16 was used to identify functional similarities between *Arabidopsis* RAD16s and yeast RAD16 proteins. Our results show that, despite (400) million years of evolutionary separation between plants and fungi, extensive homology exists between these proteins [33] (Figure S1). The protein encoded by At1g05120 is 40% identical to both ScRAD16 and SpHRP16, whereas the protein encoded by At1g02670 is 34% and 35% identical to the same proteins (respectively; Table S2); thus, we refer to At1g05120 and At1g02670 as AtRAD16 and AtRAD16b, respectively. AtRAD16 and AtRAD16b are 61% identical at the amino acid level. In agreement with the homology of these sequences, the Princeton Protein Orthology Database groups At1g05120 and At1g02670 with *S*. *cerevisiae* RAD16 and *S*. *pombe* RHP16 in several phylogenic trees [34].

**Figure 1:**
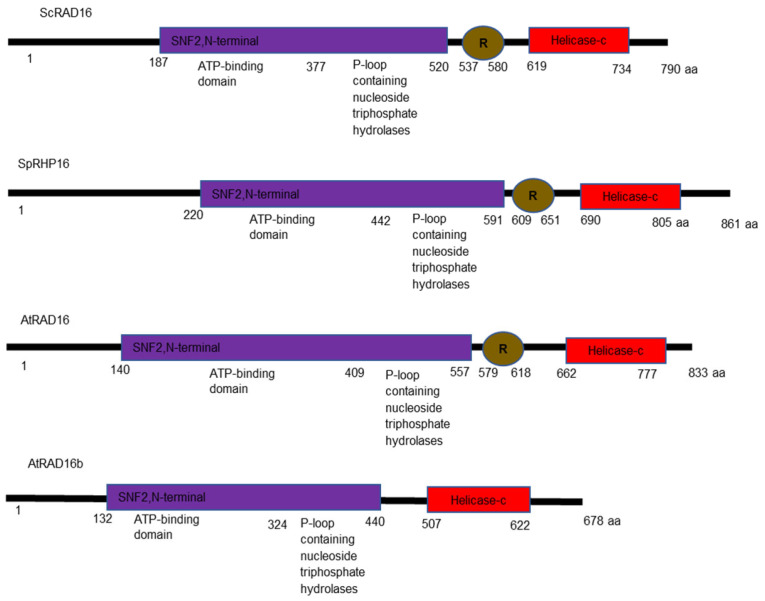
Illustration of the conserved domains and their organization in yeast RAD16 proteins and their *Arabidopsis* homologs. The SNF2, N-terminal helicase ATP binding domain is indicated in purple, R represents the C3HC4-zinc finger ring-type and indicated in brown, followed by the helicase-C terminal domain in red.

Our Pfam domain analysis shows that ScRAD16 has ATPase domains, the C3HC4-Ring domain, and the C-terminal helicase domain [35]. In ScRAD16, the ATPase domains and the C3HC4-Ring domain are required for efficient DNA repair following UV damage [36]. These functional domains exist in both *S*. *pombe* RAD16 and AtRAD16; however, AtRAD16b lacks the C3HC4-Ring domain (Figure 1).

### 3.2. Arabidopsis RAD16s Are Expressed during Development and in Response to UV Radiation

AtGenExpress data was used to examine the mRNA abundance levels of *Arabidopsis RAD16* and *RAD16b* throughout the growth, as well the expression in response to UV radiation [37]. These data show that both *AtRAD16* and *AtRAD16b* are expressed throughout development, but *AtRAD16* is expressed at higher levels than *AtRAD16b* during all developmental stages (Figure S2). Public gene expression data [38] also indicated that UV-B treatment induces the expression of *AtRAD16b* in aerial tissue > 3-fold after both 30 min and six hours treatments, while *AtRAD16b* levels drop slightly in roots under these experimental conditions (Figure S3). In contrast, *AtRAD16* level does not appear to be significantly induced in either roots or aerial tissues following UV-B treatment (Figure S3). 

### 3.3. Arabidopsis rad16 and rad16b Mutants Exhibit Increased UV Sensitivity

**Figure 2:**
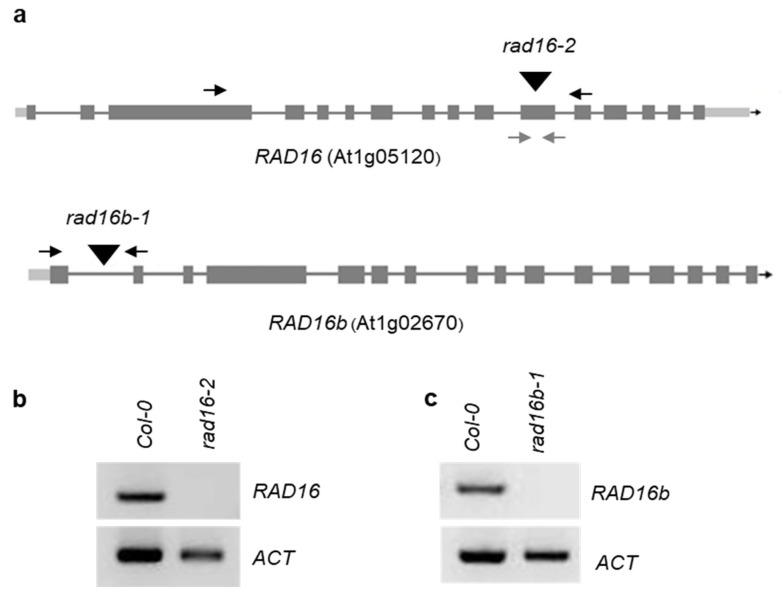
*Arabidopsis RAD16* homologues null alleles. (**a**) Gene structures of *RAD16* (At1g05120) and *RAD16b* (At1g02670). Introns are shown as lines and exons as boxes. TDNA insertion lines *rad16-2* (SALK_130522.34.85x) and *rad16b-1* (SALK_147762), sites of T-DNA insertions are shown as triangles, primer pairs used in semi-quantitative and quantitative RT-PCR are shown in black and grey arrows, respectively. (**b**) Semi-quantitative RT-PCR showing *RAD16* expression in the wild type (Col-0) and *rad16-2*. (**c**) Semi-quantitative RT-PCR showing *RAD16b* expression in the wild type (Col-0) and *rad16b-1*. ACTIN was used as a control.

The role of *S*. *cerevisiae* RAD16 and *S*. *pombe* RAD16 in yeast GG-NER has been reported [36,39], and the *S*. *cerevisiae rad16* mutant was found to exhibit a UV sensitive phenotype [40]. In this study, our protein sequence analysis results provide evidence that *Arabidopsis* RAD16 proteins are true homologs of cerevisiae RAD16 and *S*. *pombe* RAD16, suggesting a possible conserved role of *Arabidopsis* RAD16s in plant GG-NER and/or UV tolerance. Thus, we sought to perform UV sensitivity assays to examine the effects of *Arabidopsis* RAD16 loss of function on plant UV sensitivity. Two T-DNA insertion mutants were ordered from ABRC. The T-DNA insertion line used for *Atrad16* (SALK_130522.34.85x, rad16-2) is in the eleventh exon, whereas the insertion line used for *Atrad16b* (SALK_147762, rad16b-1) is in the first intron (Figure 2a). Both *Atrad16*-*2* and *Atrad16b*-*1* loss of function alleles are RNA nulls (Figure 2b,c).

**Figure 3:**
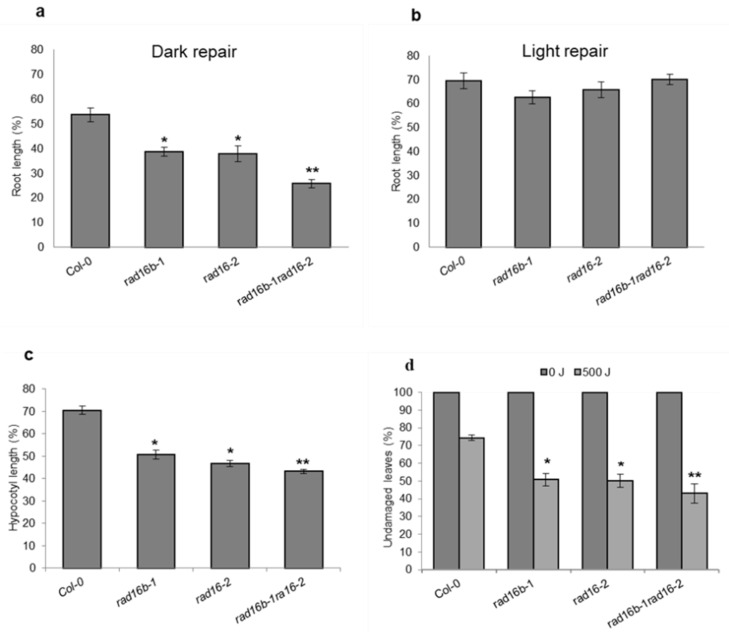
*Arabidopsis rad16* and *rad16b* single and double null mutants exhibit a UV sensitive phenotype that is dark-specific. (**a**) Relative root length of single and double mutant seedlings exposed to1000 J UV-C radiation and incubated in the dark for two days (*n* = 40). (**b**) Relative root length of single and double mutant seedlings exposed to1000 J UV-C radiation and incubated in the light for two days (*n* = 40). (**c**) Relative hypocotyl length of single and double mutant seedlings exposed to 1000 J UV-C radiation and incubated for three days in the dark (*n* = 40). (**d**) Percentage of undamaged leaves exposed to 500 J UV-C irradiation, incubated in the dark for three days, followed by four days incubation at (16h light/8h dark; *n* = 12). For (**a**–**c**), root and hypocotyl growth are presented as the treated relative to non-treated controls. Values are means ± SE, * = *p* ≤ 0.05 of single mutant vs wild type, and ** = *p* ≤ 0.05 of double mutant vs. *rad16-2*.

UV sensitivity assays were performed using *Atrad16*-*2* and *Atrad16b*-*1* single and double mutants. Both single and double mutants exhibited increased seedlings and adult UV sensitivity. Following a 1000 J m^−2^ UV treatment, four day-old seedlings exhibited a significant reduction in root and hypocotyl growth of both single mutants relative to wild-type, and double mutant relative to *Atrad16*-*2* (Figure 3a,c). Mutant adult plants also exhibited a UV sensitive phenotype as indicated by a significant increase in leaf damage in both single mutants relative to wild type, and the double mutant relative to *Atrad16*-*2* (Figure 3d and Figure S4). Thus, *Atrad16* and *Atrad16b* single and double loss of function mutant seedlings and adults are sensitive to UV radiation.

### 3.4. Arabidopsis rad16 and rad16b UV Sensitivity Is Dark-Specific

There are two DNA repair mechanisms in plants: light repair via photolyase, and dark repair via NER. We expected *Arabidopsis* RAD16 to be involved in NER. Thus, in order to confirm that *Atrad16* UV sensitivity is dark specific, after UV irradiation, we incubated one set of the UV-treated seedlings in the dark (to avoid photolyase activity) and the other set in the light. The seedlings that were incubated in the dark after UV treatment showed a significant decrease in the root length for both single and double mutants (Figure 3a). However, the seedlings of both single and the double mutant that were incubated in light after UV treatment did not exhibit any significant decrease in the root length compared to the wild type (Figure 3b), suggesting that *Atrad16* and *Atrad16b* UV sensitivity is dark repair specific, consistent with its hypothesized role in NER dark repair.

### 3.5. Lacking either AtRAD16 or AtRAD16b Results in Early Flowering Time and Short Silique Length

Based on the observation that *Arabidopsis RAD16* and *RAD16b* are expressed throughout plants development, we sought to study the role of *AtRAD16* genes in plant growth and development. We examined the effect of *Atrad16* and *Atrad16b* single and double loss of function mutants on adult growth parameters such as flowering time (in days to bolting and leaf count), height, apical dominance, silique length, and rosette width. Both *Atrad16*-*2* and *Atrad16b*-*1* single mutants showed early flowering time with respect to both days and the number of leaves. Both mutant lines also exhibited a short silique length phenotype compared to wild type (*Col*-*0*; Figure S5a,b,e). These results suggest the involvement of *Arabidopsis* RAD16s in the regulation of important growth parameters.

### 3.6. AtRAD16 Overexpression Rescues the UV-Sensitivity and Adult Developmental Phenotypes Exhibited by Atrad16 Null Mutant and Increases UV Tolerance 

**Figure 4:**
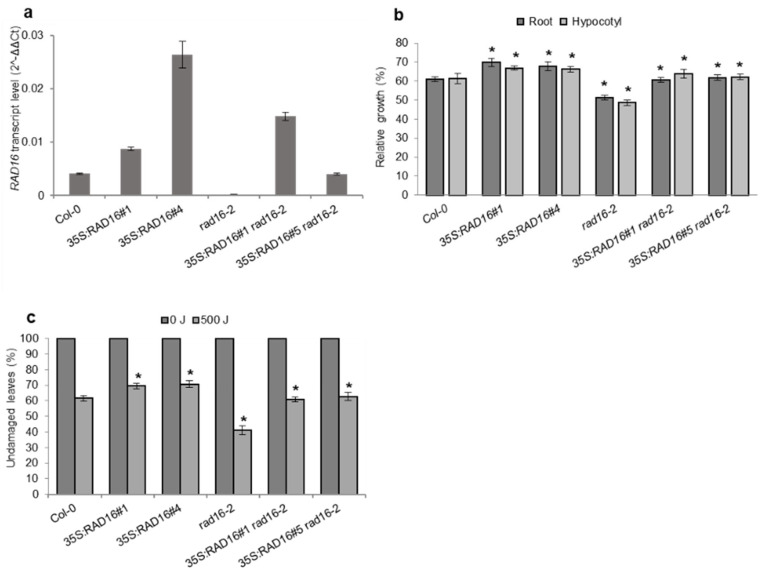
*Arabidopsis* RAD16 overexpression complements adult and seedling UV sensitive phenotypes and increases UV tolerance. (**a**) *RAD16* transcript level in Col-0 (wild type), *35S: RAD16*, *rad16-2*, and *35S: RAD16 rad16-2*. The *EF1α* gene was used to normalize the values. Error bars represent SE of the mean. (**b**) Lengths of root and hypocotyl in seedlings incubated in the dark for three days following 1000 J UV-C treatment, for each genotype, data are shown as relative to non-treated control (*n* = 40). (**c**) Percentage of undamaged leaves exposed to 500 J UV-C radiation, incubated in dark conditions for three days, followed by four days incubation at 16h light/8h dark (*n* = 12). For (**b**) and (**c**), values are means ± SE, * = *p* ≤ 0.05 of *35S: RAD16* and *rad16-2* vs. Col-0, and *35S: RAD16 rad16-2* vs. *rad16-2*.

**Figure 5:**
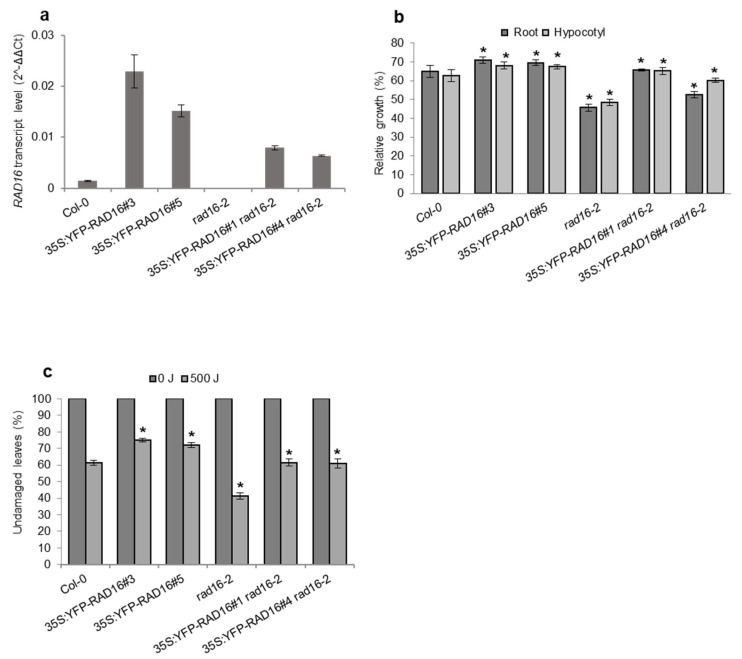
*Arabidopsis* YFP-RAD16 overexpression complements adult and seedling UV sensitive phenotype and increases UV tolerance (**a**) *RAD16* transcript level in Col-0 (wild type), *35S:YFP-RAD16*, *rad16-2*, and *35S:YFP-RAD16 rad16-2*. The *EF1α* gene was used to normalize the values. Error bars represent SE of the mean. (**b**) Lengths of root and hypocotyl in seedlings incubated in the dark for three days following 1000 J UV-C treatment, for each genotype, data are shown as relative to non-treated control (*n* = 40). (**c**) Percentage of undamaged leaves exposed to 500 J UV-C radiation, incubated in dark conditions for three days, followed by four days incubation at 16h light/8h dark (*n* = 12). For (**b**,**c**), values are means ± SE, * = *p* ≤ 0.05 of *35S: RAD16* and *rad16-2* vs. *Col-0*, and *35S: RAD16 rad16-2* vs. *rad16-2*.

To verify that the observed UV-sensitive phenotypes in both seedlings and adults and the early flowering time and short silique length phenotypes in adults were due to null insertion mutations in *Atrad16*, we carried out genetic complementation experiments. Wild-type *AtRAD16* was cloned into two overexpression vectors: 35S:RAD16, which produces a tagless version of AtRAD16; and 35S:YFP-RAD16, which produces a AtRAD16 product with an N-terminal YFP tag. In both cases, ubiquitous overexpression is driven by the CaMV 35S promoter. These constructs were transformed into the *Atrad16*-*2* null background using Agrobacterium mediated transformation. Tagless AtRAD16 overexpression lines 5S:RAD16 in the *Atrad16*-*2* background rescued seedlings and adults UV sensitivity exhibited by the *Atrad16*-*2* null mutant (Figure 4b,c). Similarly, YFP-tagged AtRAD16 overexpression lines 35S:YFP-RAD in the *Atrad16*-*2* background rescued seedlings and adults UV sensitivity exhibited by the *Atrad16*-*2* loss of function mutant (Figure 5b,c). These overexpression lines also rescued the early flowering time and short silique length in adult *Atrad16*-*2* plants (Figures S6a,b,e and S7a,b,e). These results indicate that the UV sensitivity and adult developmental phenotypes exhibited by the *Atrad16* loss of function mutant are due to a defect in *AtRAD16* gene, and it is unlikely that the YFP tag disrupts AtRAD16 function. 

To examine the effects of AtRAD16 overexpression on seedlings and adults’ UV tolerance, both 35S:RAD16 and 35S:YFP-RAD16 overexpression constructs were transformed into Col-0. Both the tagless and YFP-tagged AtRAD16 overexpression in the wild type background exhibited a significant increase in seedling and adult UV tolerance compared to the wild type (Figure 4b,c and Figure 5b,c). The expression level of *AtRAD16* in all the transgenic plants (tagless and YFP-tagged RAD16 overexpression lines) was higher than that in wild type plants (Figure 4a and Figure 5a). These results indicate that AtRAD16 overexpression is sufficient to increase UV tolerance in multiple plant late stages, and that the YFP epitope tag did not disrupt AtRAD16 function.

### 3.7. Arabidopsis RAD16 Exhibits Nuclear Localization That Is Not Changed by UV Treatment 

**Figure 6:**
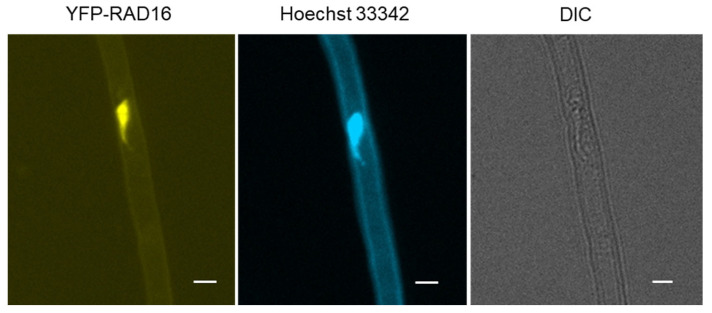
*Arabidopsis* YFP-RAD16 localizes in the nucleus. AtRAD16 localization in root hair of YFP-tagged RAD16 seedlings grown in the dark for three days was examined under the Zeiss AXIO Imager Microscope. YFP fluorescence, Hoechst 33342 dye, and differential interference contrast (DIC) are shown separately. The scale bar represents 20 μm.

**Figure 7:**
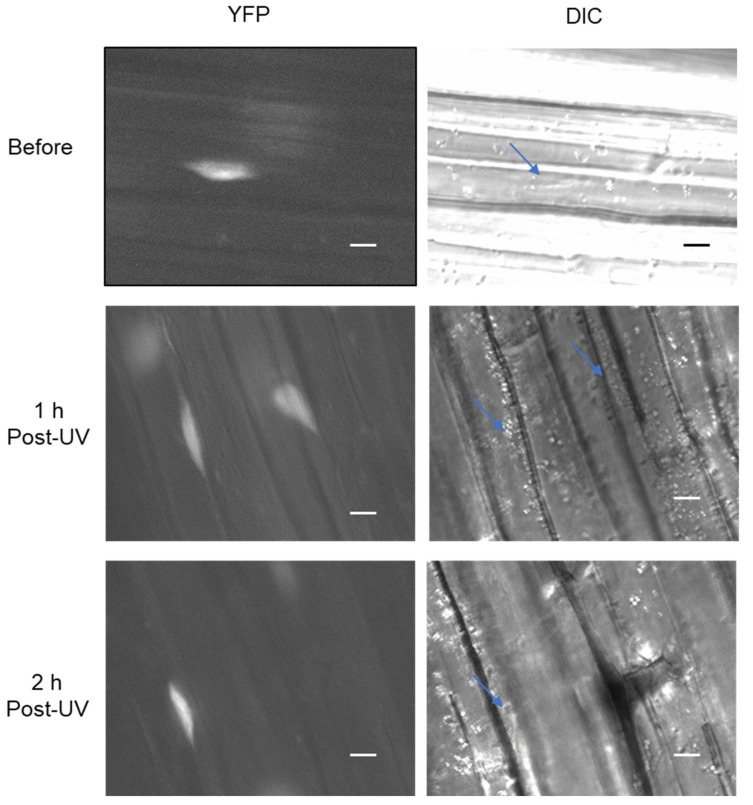
UV treatment does not alter *Arabidopsis* RAD16 nuclear localization. AtRAD16 localization in hypocotyl cells of YFP-tagged RAD16 seedlings grown in the dark for three days was examined before and after one and two hours following UV treatment for YFP fluorescence (blue arrows indicate the localization of RAD16 protein), and differential interference contrast (DIC). The scale bar represents 10 μm.

*Arabidopsis* NER proteins are either localized to the nucleus or are relocalized to the nucleus from the cytoplasm in response to DNA damage [18]. Given that *Arabidopsis* RAD16 is expected to be involved in NER, we sought to examine AtRAD16 cellular localization before and after UV treatment. In order to examine AtRAD16 cellular localization, 35S:YFP-RAD16 #3 overexpression line in Col-0 background was examined for YFP fluorescence. Non-UV treated plants exhibited YFP fluorescence in the nucleus, and no YFP fluorescence was observed in the cytosol of YFP-tagged RAD16 plants. This observation suggests that YFP tagged RAD16 is localized exclusively throughout the nucleus, as verified with Hoechst staining (Figure 6). In order to examine the effects of UV radiation on AtRAD16 nuclear localization, cellular localization of the same YFP tagged overexpression line was examined following UV treatment. AtRAD16 nuclear localization was not altered following UV treatment (Figure 7). 

### 3.8. Arabidopsis RAD16 Physically Interacts with Arabidopsis RAD7a and RAD7c

The Arabidopsis Interaction Viewer identified two *Arabidopsis* RAD7 homologues: RAD7a encoded by At2g06040 and RAD7b encoded by At4g15475. It also identified an ELC homologue encoded by At5g59140 (AtELOC) as potential interaction partners of AtRAD16, based on the interactions of the homologous proteins in yeast [41]. The Genemania prediction server [42], which is used to identify the potential interaction partners of proteins based on publicity available databases, also predicts interactions between AtRAD16 and each of AtRAD7a, At5g21900 (RAD7c), and AtELOC. In order to determine whether *Arabidopsis* RAD16 interacts as the predicted yeast model, or if it has additional interaction partners, we sought using yeast two-hybrid analysis to examine physical interactions of AtRAD16 with other AtRAD7/16 complex proteins (AtRAD7a, AtRAD7b, AtRAD7c, AtELOC), as well as with other GG-NER components, such as AtRAD4, AtDDB2, and AtRAD23.

**Figure 8:**
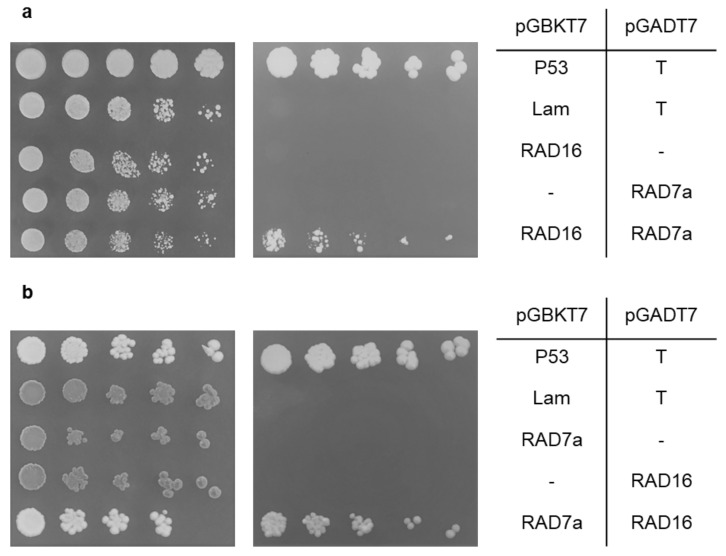
Yeast two-hybrid screening for *Arabidopsis* RAD16/RAD7a interaction. RAD16 interacts with RAD7a. Each of RAD16 and RAD7a were cloned into pGBKT7 (bait) and pGADT7 (prey) vectors and transformed into haploid yeast cells, then mated. Five-fold dilutions of the mated diploid strain with a starting concentration of 0.2 optical density at 600 nm. (**a**) RAD16 bait/RAD7a prey and (**b**) RADa bait/RAD16 prey were spotted on (-leu -trp) non-selective plates (**left**) and on selective plates (-leu –trp –ade -his) (**right**). P53/T and Lam/T are the positive and negative controls, respectively, as P53 interacts with T, whereas Lam does not interact with T.

**Figure 9:**
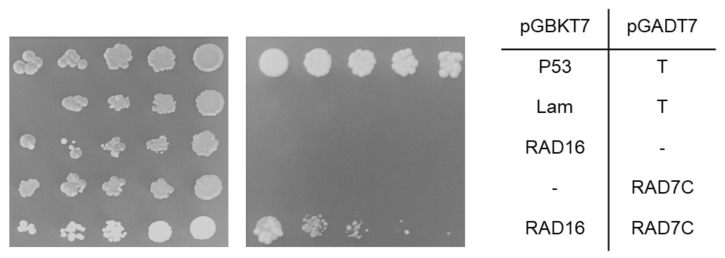
Yeast two-hybrid screening for *Arabidopsis* RAD16/RAD7c interaction. RAD16 interacts with RAD7c. *RAD16* and *RAD7c* were cloned into pGBKT7 (bait) and pGADT7 (prey) vectors (respectively) and transformed into haploid yeast cells, and then mated. Five-fold dilutions of the mated diploid strain with a starting concentration of 0.2 optical density at 600 nm.; RAD16 bait/RAD7c prey were spotted on (-leu -trp) non-selective plates (**left**) and on selective plates (-leu –trp –ade -his) (**right**). P53/T and Lam/T are the positive and negative controls respectively, as P53 interacts with T, whereas Lam does not interact with T.

In the yeast two-hybrid systems, the interaction was tested by growing diploid stains on quadruple drop-out selective medium (-leu –trp –his –ade). The diploid yeast strain that has *Arabidopsis RAD16* as bait and *AtRAD7a* as a prey grew on the selective media, indicating that AtRAD16 interacts with AtRAD7a and induces the expression of the *ADE2* and *HIS3* reporters which allow the diploid yeast to grow on the selection media. This interaction was seen between AtRAD16 bait/AtRAD7a prey and vice versa (Figure 8a,b). Moreover, AtRAD16 and AtRAD7a did not interact with themselves (Figure S8a,b). AtRAD16 pGBKT7 (bait) was also found to interact with AtRAD7c pGADT7 (prey; Figure 9). However, we could not examine the interaction between AtRAD16 pGADT7 (prey) and AtRAD7c pGBKT7 (bait) as AtRAD7c pGBKT7 (bait) was found to interact with the pGADT7 (prey) empty vector. No interaction was detected between *Arabidopsis* RAD16 and each of AtRAD7b, AtRAD4, AtDDB2, AtELOC, or AtRAD23b (Figures S9–S13).

### 3.9. Arabidopsis rad16 rad7a and rad7a rad7c Double Null Mutant Plants Are More Sensitive to UV Radiation Than Single Mutants

**Figure 10:**
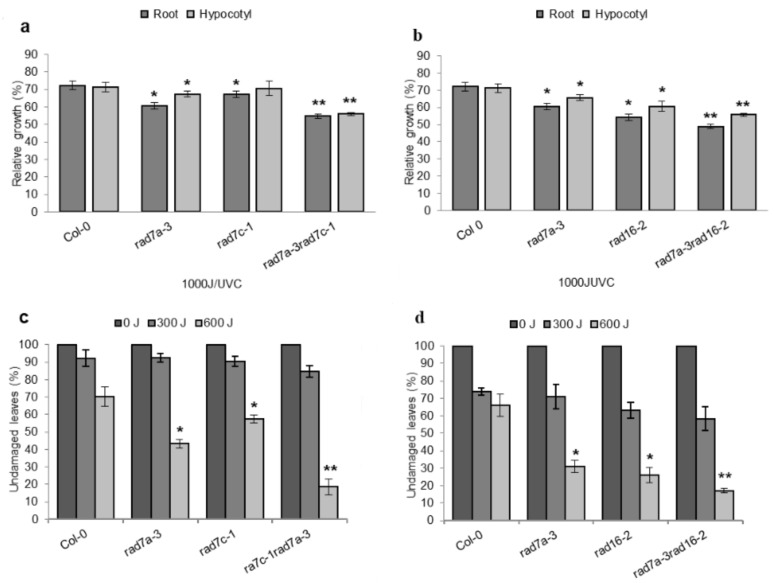
Double loss of function mutation in *Arabidopsis RAD7a*/*RAD7c* and *RAD7a*/*RAD16* increase UV sensitivity. Lengths of root and hypocotyl in *rad7a rad7c* (**a**) and *rad7a rad16* (**b**) seedlings incubated in the dark for three days following 1000 J UV-C treatment. For each genetype, data are shown as relative to non-treated control (*n* = 40). Percentage of undamaged leaves in *rad7a rad7c* (**c**) and *rad7a rad16* (**d**) adults exposed to varying doses of J UV-C radiation, incubated in dark conditions for three days, followed by four days incubation at 16 h light/8 h dark (*n* = 12). Values are means ± SE, * = *p* ≤ 0.05 of single mutant vs. wild type, and ** = *p* ≤ 0.05 of double mutant vs. single mutant.

The results of this study show that a defect in the *Arabidopsis RAD16* gene results in increased UV sensitivity (Figure 3a,c,d), and AtRAD16 physically interacts with AtRAD7a and AtRAD7c providing evidence that *Arabidopsis* RAD7/16 complex is required for plant UV tolerance. Therefore, we decided to examine the genetic interactions within the AtRAD7/16 complex. To examine the interaction within AtRAD7/16 complex, we generated *Atrad7a Atrad7c* and *Atrad16 Atrad7a* double mutants. Then, we assessed the effects of double loss of function on *Arabidopsis* UV tolerance and growth. Both *Atrad7a Atrad7c* and *Atrad16 Atrad7a* double mutants exhibited a significant decrease in roots and hypocotyls length compared to *Atrad7a* and *Atrad16* single mutants (Figure 10a,b). Also, *Atrad7a Atrad7c* and *Atrad16 Atrad7a* adult plants exhibited a significant increase in the percentage of UV-damaged leaves compared to *Atrad7a* and *Atrad16* single mutants (Figure 10c,d). The increased UV sensitivity exhibited by both *Atrad7a Atrad7c* and *Atrad16 Atrad7a* may reflect the additive UV sensitivity exhibited by single mutants in each double. Compared to single mutants, neither *Atrad7a Atrad7c* nor *Atrad16 Atrad7a* has a significant effect on adult growth parameters, such as flowering time in days and leaf counts, height, apical dominance, silique length, and rosette width. 

### 3.10. Lacking Both Arabidopsis RAD16 and RAD23b Results in Embryo Lethality

**Figure 11:**
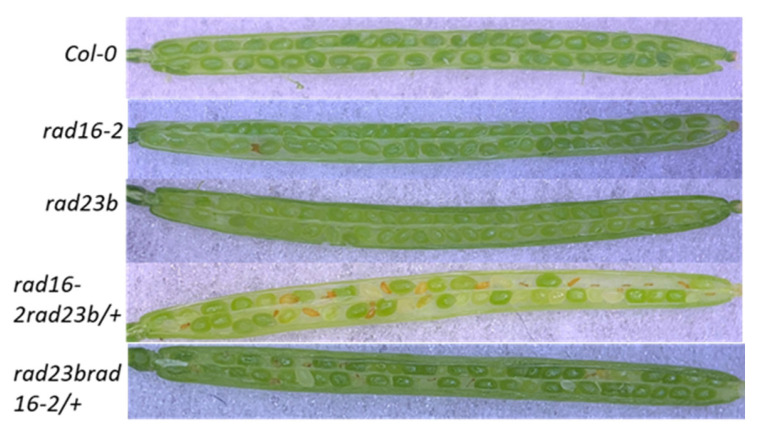
Silique phenotypes: Images of siliques obtained from self-pollinated wild type (Col-0), *rad16-2*, *rad23b*, *rad16-2rad23b/+*, and *rad23brad16-2/+*. Abnormal ovules are visible in the heterozygous line.

To examine the genetic interaction between AtRAD7/16 complex and *Arabidopsis* NEF2 complex (RAD23/RAD4), which is also involved in DNA damage recognition and facilitating the recruitment of NER components to the damaged site. We attempted to generate *Atrad16Atrad23b* double mutants. However, no *Atrad16Atrad23b* homozygous double mutants were ever recovered from this cross. We were able to grow plants that were heterozygous mutant for one gene and homozygous for the other (*Atrad16 Atrad23b*/+, and *Atrad16*/+ *Atrad23b*). Siliques of these lines were screened for the presence of defective seeds. Approximately 22% of the seeds were deformed, suggesting that *Atrad16Atrad23b* double mutants result in embryo lethality (Figure 11 and Table 1) Thus, we hypothesized that the number of deformed seeds in each of each of *Atrad16Atrad23b*/+ and *Atrad16*/+ *Atrad23b* will be 25% of the total number of seeds. To validate our hypothesis, we performed a Chi-square statistical analysis. The Chi-square statistical values were as follows: *Atrad16Atrad23b*/+, *X^2^* = 1.5122x10^−28^, df = 1, *p*-value = 1 and for *Atrad16*/+ *Atrad23b*, *X^2^* = 2.003, df = 1, *p*-value = 0.157. The chi-square calculated values are less than the critical values that are required to reject our stated hypothesis. 

### 3.11. RAD7/16 Damage Recognition Module Makes a Significant Contribution to Arabidopsis NER Compared to the DDB1/2 Module

**Figure 12:**
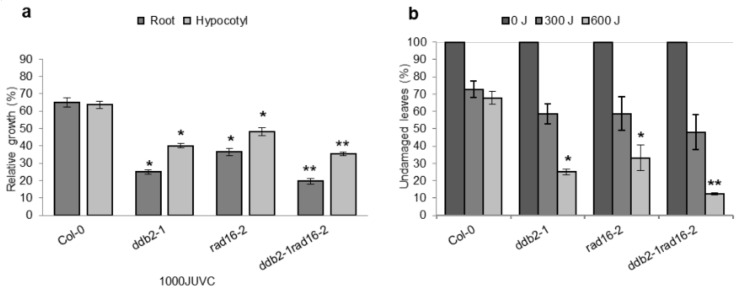
Double loss of function mutation in *Arabidopsis DDB2/RAD16* increases UV sensitivity. (**a**) Root and hypocotyl lengths of single and double-mutants seedlings incubated in the dark for three days following 1000 J UV-C treatment. For each genotype, data are shown as relative to non-treated control (*n* = 40). (**b**) Percentage of undamaged leaves in adult plants exposed to varying doses of UV-C radiation, incubated in dark conditions for three days, followed by four days incubation at 16h light/8h dark (*n* = 12). Values are means ± SE, * = *p* ≤ 0.05 of single mutant vs wild type, and ** = *p* ≤ 0.05 of double mutant vs ddb2-1mutant.

*Arabidopsis* contains both mammalian and yeast homologs that participate in DNA damage recognition, which provides a unique opportunity to clarify the individual contributions and the genetic interactions between DDB1/2 and RAD7/16 (homologs of mammalian and yeast GG-NER damage recognition factors, respectively) in a single system. To do so, we generated an *Atrad16Atddb2* double loss of function mutant and assessed the effects of lacking both potential damage recognition pathways on *Arabidopsis* UV tolerance. The double mutant exhibited enhanced UV sensitivity compared to single mutants in seedlings and adults, following treatment with 1000 and 600 J, respectively (Figure 12). This suggests that the yeast-like GG-NER damage recognition factors RAD7/16 make a significant contribution to wild-type UV tolerance and/or GG-NER compared to the mammalian-like GG-NER damage recognition factors DDB1/2. With respect to plant developmental phenotypes, we did not observe any significant differences between the double and single mutants.

### 3.12. The RAD7/16 Pathway Makes a Significant Contribution to Arabidopsis NER Compared to the CSB Pathway 

**Figure 13:**
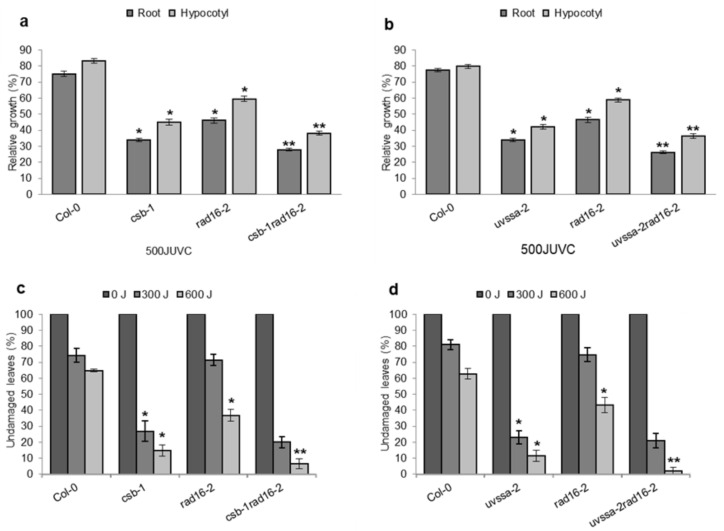
Double loss of function mutation in *Arabidopsis CSB/RAD16* and *UVSSA/RAD16* increases UV sensitivity. Lengths of root and hypocotyl in (**a**) *csb rad16* and (**b**) *uvssa rad16* seedlings incubated in the dark for three days following 500 J UV-C treatment. For each genotype, data are shown as relative to non-treated control (*n* = 40). Percentage of undamaged leaves in (**c**) *csb rad16* and (**d**) *uvssa rad16* adults exposed to varying doses of J UV-C radiation, incubated in dark conditions for three days, followed by four days incubation at 16 h light/8 h dark; *n* = 12). For (**a**–**d**), values are means ± SE, * = *p* ≤ 0.05 of single mutant vs. wild type, and ** = *p* ≤ 0.05 of double mutant vs. single mutants.

In the *Arabidopsis* genome, homologs of both mammalian TC-NER proteins and yeast GG-NER proteins have been identified. In this study, we characterized the role of the yeast GG-NER homologs (RAD7/16 complex) in plant GG-NER. The role of the mammalian TC-NER homologs (CSB/UVSSA) has been previously studied [25,43]. Thus, we sought to assess the relative contribution of the yeast GG-NER homologs (RAD7/16) and the mammalian TC-NER homologs (CSB/UVSSA) to *Arabidopsis* UV tolerance. We generated double mutants with the TC-NER specific components *AtCSB* and *AtUVSSA* and *AtRAD16*, and assessed the genetic effects of simultaneous null mutations in these complexes on UV tolerance. Both the *Atrad16*-*2Atcsb*-*1* and the *Atrad16*-*2Atuvssa*-*2* double mutants exhibited increased UV sensitivity in seedlings; this sensitivity resulted in reduction in roots and hypocotyl length following 500 J UV treatment compared to wildtype (Figure 13a,b). Also, *Atrad16*-*2Atcsb*-*1* and *Atrad16*-*2Atuvssa*-*2* exhibited an increase adult UV sensitivity (compared to the single mutants (Figure 13c,d). These results indicate that the RAD7/16 pathway makes a significant contribution to the dark repair process compared to the CSB pathway. With respect to plant developmental phenotypes, we did not observe any statistical differences between the single and double mutants. 

## 4. Discussion

In mammals, GG-NER is initiated by the damage recognition complex DDB1/2. In the yeast *S*. *cerevisiae*, GG-NER is initiated by the RAD7/16 complex, which performs many of the same functions as DDB1/2. Like the mammalian complex, the RAD7/16 complex is an E3 ubiquitin ligase complex that causes chromatin modification, ubiquitinating ScRAD4, as well as subsequent recruitment of conserved downstream core NER activities [16]. *S*. *cerevisiae* RAD16 and *S. pombe* RAD16 homolog (rhp16), as members of this complex, are required for GG-NER [39,44,45]. In plant systems, the DDB1/2 complex have been shown to be necessary for GG-NER [22,23].

In this study, we characterized the roles of the two *Arabidopsis* RAD16 homologs in plant UV tolerance and growth. Findings from this study have demonstrated for the first time the involvement of AtRAD16 and AtRAD16b in plant UV tolerance. We also show the involvement of these homologs in plant growth and development. 

### 4.1. Suggested Conserved Function of Arabidopsis RAD16s in DNA Repair

Our protein sequence analysis results show that AtRAD16 and AtRAD16b are more related to each other than to yeast RAD16 (Table S2), suggesting that both *AtRAD16* and *AtRAD16b* have evolved through gene duplication. Protein functional analysis shows that ScRAD16 has SNF2 related domain and the C3HC4-Ring domain (Figure 1). AtRAD16 also has these two domains. In ScRAD16, the ATPase activity of the SNF2 related domain and the E3 ubiquitin ligase activity of the ring domain are required for efficient GG-NER, since both affect RAD16 distribution during GG-NER. Individually mutating these domains results in a mild UV sensitivity and reduction in the DNA repair rates. Inactivation of both domains results in increased UV sensitivity resembles that exhibited by the *scrad16* null mutant [36]. Thus, both domains are required for efficient GG-NER in yeast. AtRAD16b lacks the ring domain, but it has the SNF2 domain (Figure 1); therefore, we expect AtRAD16b to play a partial redundant function with AtRAD16 in plant GG-NER.

The GG-NER components in mammals and yeast, DDB1/DDB2/CUL4 and RAD7/RAD16/ELC/CUL3, have E3 ubiquitin ligase activities [12,17]. Thus *S*. *cerevisiae* Rad7/16 complex interacts with CUL3 and ELC, forming a CUL3-type ubiquitin ligase complex that is involved in NER [46]. Since AtRAD16 also possesses the ring domain, it is possible that AtRAD16 has the ability to form a Cullin-based E3 ubiquitin ligase complex. If so, it will be compelling to identify the components of the E3 ubiquitin ligase complex that AtRAD16 might form.

In this study, AtRAD16 was found to physically interact with AtRAD7. Thus, it is speculated that RAD7/RAD16/ELC/CUL1 functions as an E3 ubiquitin ligase that is involved in UV tolerance and development. These results provide evidence that *Arabidopsis* RAD16 homologs may have a conserved role in plant GG-NER.

### 4.2. Arabidopsis RAD16s Are Required for GG-NER (Dark Repair)

The role of RAD7/16 damage recognition factors in GG-NER has been studied in the yeast system [47]. *Arabidopsis* has two homologs of yeast RAD16 (AtRAD16 and AtRAD16b) and, in this study, we found these homologs to be involved in *Arabidopsis* GG-NER/or UV tolerance. Both *Atrad16* and *Atrad16b* single and double null mutants exhibited a significant increase in seedling and adult UV sensitivity (Figure 3a,c,d). We found that the UV sensitivity exhibited by both null alleles is dark-specific (Figure 3a,b), which indicates that *Arabidopsis* RAD16 and RAD16b are involved in the dark repair pathway (GG-NER), rather than the light repair pathway of UV induced DNA photoproducts. Even though AtRAD16b was not predicted to interact with other AtRAD7 complex members [41,42], *AtRAD16b* expression was found to be induced in response to UV-B radiation [38] in the wild type plants. Thus, AtRAD16b may perform a partially redundant function with AtRAD16, and this may account for the additive increased UV-sensitivity observed in the *Atrad16Atrad16b* double mutant, compared to both the *Atrad16* and *Atrad16b* single mutants (Figure 3a,c,d). Consistent with our results, *S*. *cerevisiae rad16* loss of function mutant exhibits increased UV sensitivity [40], and ScRAD16 was found to contribute to GG-NER [44]. Similarly, the *S*. *pombe* RAD16 homologue (rhp16) is also required for GG-NER [39,45]. The UV-sensitivity of *Arabidopsis rad16* and *rad16b* single and double null mutants provides further evidence in support of the involvement of AtRAD16 and AtRAD16b in GG-NER (dark repair).

### 4.3. Regulation of Developmental Processes

*S*. *pombe* RAD16 is required for normal chromosome segregation, and the loss of function of ScRAD16 resulted in a reduction in spore viability. In yeast vegetative cells, RAD16 is required for maintaining genome stability [45]. In this study, we have shown the involvement of the *Arabidopsis* RAD16 homologs in plant growth and development. *Atrad16* and *Atrad16b* single mutants showed early flowering time and short silique length compared to wild type (Figure S5a,b,d). Additionally, AtRAD16 overexpression rescued the early flowering time and short silique length exhibited by the *Atrad16* loss of function mutant (Figures S6a,b,d and S7a,b,d). These results suggest that AtRAD16s are important for flowering time regulation and silique development. Interestingly, *Atrad16* loss of function mutants exhibited the opposite flowering time phenotype (early) of that previously exhibited by the *Atcul3* null mutant (late; Figure 4a,b) [48]. This may indicate that AtRAD16s are negative regulators of AtCUL3 activity. The *Arabidopsis* genome has two CUL3 proteins: CUL3A and CUL3B. CUL3 is a member of the CULLIN family of proteins which act as the molecular scaffold in E3 ubiquitin ligase complexes. Cullin-based E3 ubiquitin ligases are involved in many developmental processes. For example, CUL3/LRB 1&2 play a role in light signaling including flowering time regulation [49] to control flowering time. Double loss of function mutant *Atcul3Atcul3b* is embryo lethal. Whereas a double mutant *cul3a*-*3cul3b*-*1*, which demonstrates a partial *CUL3a* loss of function and complete *CUL3b* loss of function, has been reported to show a reduced rosette diameter, in addition to delayed flowering time, demonstrating its important in plant development [50]. Similarly, *Atrad16* and *Atrad16b* single mutants showed early flowering time and short silique length compared to wild type. Thus, the role of AtRAD16 and AtRAD16b is not limited to DNA repair only, but it extends to the regulation of important growth parameters.

### 4.4. Overexpression of NER Components 

Overexpression of AtRAD16 in wild type plants significantly increased adults and seedlings UV tolerance compared to the wild type. Moreover, overexpression of AtRAD16 in *Atrad16*-*2* null mutant complemented the UV sensitivity exhibited by *Atrad16*-*2* mutant (Figure 4b,c) and (Figure 5b,c). In *S*. *cerevisiae*, a purified RAD7/16 complex from cells overexpressing both proteins was found to bind to damaged DNA and promote the excision of damaged DNA fragment in vitro [51]. Also, *ScRAD16* expression restored the UV-sensitive phenotype exhibited by the yeast *rad16* mutant [40]. Thus, our results suggest that *Arabidopsis* RAD16 contributes to plant UV tolerance and or/GG-NER, and it is limiting. Consistent with our results, the overexpression of GG-NER proteins such as AtRAD4, AtRAD7, AtCEN2, AtDDB2, and AtDDB1a resulted in increased UV tolerance in *Arabidopsis* [18,23,24,52]. Our findings, together with the previous findings from *Arabidopsis* and yeast GG-NER studies, support our hypothesis, which states that *Arabidopsis* RAD16 is a component GG-NER. 

### 4.5. Cellular Localization of NER Components 

We analyzed AtRAD16 subcellular localization using YFP-tagged RAD16 seedlings. AtRAD16 was found to localize throughout the nucleus (Figure 6). The PSI (plant subcellular localization integrative predictor) [53] and the consensus algorithm SUBAcon [54] predict AtRAD16 to localize to the nucleus with scores of 0.959 and 1.000, respectively. Additionally, the cNLS mapper predicts AtRAD16 to have classical monopartite NLSs [55]. Thus, our AtRAD16 nuclear localization result was consistent with the results obtained by in silico prediction tools. AtRAD16 nuclear location was not affected by UV treatment (Figure 7).

AtRAD16 nuclear localization is consistent with the role of AtRAD16 in NER that takes place in the nucleus after UV damage. This nuclear localization is also consistent with the dark-specific UV sensitivity exhibited by *Atrad16* loss of function mutant (Figure 3a,c,d), and the increased UV tolerance exhibited by AtRAD16 overexpression (Figure 4b,c and Figure 5b,c), which all suggest the involvement of AtRAD16 in UV tolerance and/or GG-NER. 

Like AtRAD16, other *Arabidopsis* NER proteins—such as AtRAD7, AtRAD4, and AtDDB2—are nuclear localized [18,23,24]. GFP-tagged AtCSA, a TC-NER protein also exhibited nuclear localization [56]. However, the *Arabidopsis* homolog of mammalian NER factor CEN2 (AtCEN2) exhibited cytoplasmic localization under unstressed conditions, and is shuttled to the nucleus upon UV treatment [52]. Similarly, *Arabidopsis* DDB1 (a homolog of mammalian DDB1 damage recognition factor) was found to relocalize from the cytosol to the nucleus following UV treatment [23]. These results suggest that NER proteins that are located in the cytosol under unstressed conditions, shuttled to the nucleus following UV radiation; whereas, those that are located in the nucleus remain there. 

Consistent with these results, GFP tagged ScRAD16 was found to be localized in the nucleus. Treatment with methyl methanesulfonate and hydroxyurea, DNA damaging agents, did not change this pattern [57]. *S*. *pombe* RAD16 homolog rhp16 is also predicted to be localized to the nucleus, as predicted by the eukaryotic protein subcellular localization predictor [58].

### 4.6. Suggested Role for Arabidopsis RAD7/16 Complex in GG-NER

We used yeast two-hybrid assay to identify the proteins that physically interact with AtRAD16. Our results showed physical interactions between the following protein pairs: AtRAD16 with AtRAD7a (Figure 8), and AtRAD16 with AtRAD7c (Figure 9). This result is consistent with the Genemania prediction server, which predicts an interaction between AtRAD16 and AtRAD7a, as well as between AtRAD16 and AtRAD7c. However, no interaction was detected between AtRAD16 and AtRAD7b, and this result contradicts the AIV prediction model that predicts an interaction between AtRAD16 and AtRAD7b (Figure S9). Protein sequence analysis of *Arabidopsis* RAD7a, RAD7b, and RAD7c shows that AtRAD7a and AtRAD7c are more closely related to each other than to AtRAD7b, as they share 43% amino acid identity. However, AtRAD7b shares 27% and 24% amino acid identity with AtRAD7a and AtRAD7c, respectively, which may explain the lack of interaction between AtRAD16 and AtRAD7b. 

Consistent with our results, in *S*. *cerevisiae* an interaction between ScRAD16 and ScRAD7 has been detected in yeast two-hybrid assays [59]. ScRAD16 interacts with ScRAD7 and forms the yeast GG-NER initiation complex known as NEF4 in response to UV-damaged DNA. The interaction between ScRAD16 and ScRAD7 is required for the repair of transcriptionally inactive DNA in vivo [60]. Therefore, the interaction between AtRAD16 and AtRAD7 suggests that the yeast GG-NER initiation complex RAD7/16 is active in *Arabidopsis*.

AtRAD16 did not physically interact with each of AtELOC (Figure S12), and AtRAD23 (Figure S12). Consistent with our results, no interaction between ScRAD16 and ScELC1 or ScRAD23 has been demonstrated in yeast. Nevertheless, physical interaction between ScRAD16 and ScELC1 was determined by using co-purification techniques [17], and genetic interaction was detected between *ScRAD16* and *ScRAD23* [60]. Our results also showed genetic interaction between *AtRAD16* and *AtRAD23*. No physical interaction was detected between AtRAD16 and AtRAD4 (Figure S10). Similarly, ScRAD16 was found not to physically interact with ScRAD4 using yeast two-hybrid assay [59]. 

### 4.7. AtRAD16 and AtRAD7 Are Involved in UV Tolerance 

*Arabidopsis rad7* and *rad16* single loss of function mutants exhibited enhanced UV sensitivity in both seedlings and adults compared to the wild type (Figure 10b,d), and *Arabidopsis rad7rad16* double loss of function mutant plants are more sensitive to UV radiation than the single null mutants. Consistent with our results, loss of function studies of the yeast *S*. *cerevisiae* showed that *ScRAD7* and *ScRAD16* are required for the repair of the untranscribed regions of the genome, and loss of either gene resulted in a moderate UV sensitivity [47,60,61]. However, yeast *rad7rad16* double loss of function mutant exhibited UV sensitivity resemble that of single mutants [59,62]. Thus, our results suggest that both *Arabidopsis* RAD7 and RAD16 are required for plant UV tolerance. Moreover, the role of *Arabidopsis* RAD7and/or RAD16 extends the damage recognition pathway identified in yeast to other NER pathways. Additionally, The UV sensitivity exhibited by the *Atrad7a Atrad16* double null mutant plants resembles that exhibited by *Atra7aAtrad7c* double null mutant plants. This result may account for the partial redundancy between *AtRAD7a* and *AtRAD7c*; thus, AtRAD7c might partially complement *Atrad7a* loss of function in the *Atrad7aAtrad16* double null mutant.

### 4.8. Arabidopsis RAD16 and RAD23 Are Required for Embryo Development

We examined the genetic interaction between *AtRAD16* and *AtRAD23b* and found that loss of both *AtRAD16* and *AtRAD23b* resulted in embryo lethality. This suggests that AtRAD16 acts with AtRAD23b regulate growth and development. In the *S*. *cerevisiae*, RAD4 alone is expected to be intrinsically unstable and RAD23 binding to RAD4 was found to stabilize RAD4 levels in unstressed cells [63]. Additionally, ScRad23 is functionally redundant with one of the ScRAD16 biochemical activities [60], so it is possible that ScRAD16 is also involved in stabilizing the ScRAD4 level. In *Arabidopsis*, it is possible that both AtRAD23 and AtRAD16 are also involved in stabilizing AtRAD4 level and preventing its degradation like their yeast counterparts. Thus, loss of function of both AtRAD16 and AtRAD23b may result in AtRAD4 instability and degradation. Since the loss of function mutation of AtRAD4 was found to be lethal [24]. Our finding may indicate that the embryo lethality of the *Arabidopsis rad16rad23b* double mutant is an indirect effect of the reduced or unstable AtRAD4 levels in the double mutant. 

Alternatively, given that *Atrad23b* loss of function mutant exhibited a mild sterility defect [64] and *Atrad16* loss of function mutation resulted in short silique length and early flowering time, it is possible that both AtRAD16 and AtRAD23 are involved in the regulation of important growth parameters. Thus, the loss of both AtRAD23 and AtRAD16 results in defect in embryo development.

### 4.9. Both RAD7/16 and DDB1/2 Damage Recognition Modules Contribute to Arabidopsis UV Tolerance

The role of AtDDB2 (a homolog of the mammalian GG-NER damage recognition factor DDB2) in plant UV tolerance has been reported [65]. The *Atddb2* single null mutant exhibits increased UV sensitivity [23], and in this research, *the Atrad16* single mutant also exhibited increased UV sensitivity (Figure 3). Thus, both AtRAD16 and AtDDB2 contribute to plant UV tolerance and/or DNA repair. We also showed that the UV sensitivity exhibited *Atddb2* null mutant is more than 10% higher than the UV sensitivity exhibited *Atrad16* null mutant. In addition, the UV sensitivity of *Atrad16 Atddb2* double null mutant plants is significantly higher than that exhibited by the *Atrad16* and the *Atddb2* single null mutants (Figure 12). The enhanced UV sensitivity exhibited by *Atrad16Atddb2* double mutant reflects the additive UV sensitivity exhibited by single mutants, and also indicates that the yeast homolog (RAD7/16 complex) makes a significant contribution to plant GG-NER compared to the mammalian homolog (DDB1/2 complex). This result is consistent with our plant GG-NER model, as we expect homologs of both yeast and mammalian GG-NER damage recognition factors to contribute to plant GG-NER by forming two different complexes. The mammalian and yeast damage recognition factors, DDB1/2 and RAD7/16, share functional similarities, including the recognition of UV-induced photoproducts, and histone modification via interacting with histone acetyl transferase GCN5 and ubiquitinating conserved components XPC/RAD4. However, the mechanism that these complexes use to recognize the UV-induced photoproducts are different. RAD7/16 complex scans DNA using the RAD16 ATPase as a motor; thus, damage recognition by this complex requires ATP [66]. However, DDB1/2 complex uses jumping as a target search mechanism in an ATP-independent manner [67]. The mammalian DDB1/2 and yeast RAD7/16 damage recognition factors form two different E3 ubiquitin ligase complexes that are required for XPC and RAD4 ubiquitination, respectively. XPC ubiquitination enhances its binding affinity, whereas RAD4 ubiquitination results in its degradation [68,69]. Therefore, it is possible that DDB1/2 activity complements RAD7/16 activity. In addition, XPA/RAD14 homolog does not exist in plants; XPA/RAD14 binds the damaged site at the single-strand DNA. As a result, it is possible that plants use the mammalian and yeast damage recognition modules to counteract the absence of XPA homolog [70,71]. This work shows clearly that plants are not just green mammals; they draw on the strength of breadth of systems in a unique way.

### 4.10. Both Arabidopsis RAD16 and CSB/UVSSA Contribute to Plant UV Tolerance and CSB/UVSSA Pathway Is More Essential at Early Developmental Stages 

In *Arabidopsis*, CSB and UVSSA (the homologs of mammalian TC-NER factors), were found to be involved in TC-NER. Inactivation of either AtCSB or AtUVSSA results in increased UV sensitivity [25,43]. Findings from this study demonstrated that *AtcsbAtrad16* and *AtuvssaAtrad16* double null mutants are UV radiation sensitive compared to single mutants in both seedlings and adults (Figure 13). The adult UV sensitivity exhibited by *Atcsb Atrad16* and *AtuvssaAtrad16* mutants was more severe than the adult UV sensitivity exhibited by *rad7rad16* and *ddb2rad16* double mutants. Since *Atrad7aAtrad16* double loss of function results in ~80% leaf damage compared to ~70% and ~60% leaf damage in *Atrad7a* and *Atrad16* single null mutants, respectively (Figure 10d). *Atddb2Atrad16* double loss of function results in ~85% leaf damage compared to ~75% and ~65% leaf damage exhibited by *Atddb*-*2* and *Atrad16* single loss of function mutants, respectively (Figure 12b). Whereas *AtcsbAtrad16* and *AtuvssaAtrad16* double loss of function resulted in ~95% and ~97% leaf damage, respectively, compared to ~85% leaf damage exhibited by each of the *Atuvssa* and *Atcsb* single loss of function mutants and ~65% exhibited by the *Atrad16* single null mutant (Figure 13c,d). Similarly, the seedling UV sensitivity was observed, even with a low UV dosage (500J). The reduction in root and hypocotyl lengths exhibited by *Atcsb Atrad16* and *AtuvssaAtrad16* double loss of function following irradiating with 500 J was almost the same as the reduction exhibited by *Atrad7a Atrad16* and *Atddb2Atrad16* loss of function mutants following 1000 J UV treatment (Figure 10b, Figure 12a and Figure 13a,b). Additionally, irradiating *Atcsb Atrad16* and *AtuvssaAtrad16* double loss of function mutant seedlings with 1000 J resulted in a sever reduction in seedlings growth to a degree that prevents the detection of the seedlings’ UV sensitivity, which may indicate that the role that TC-NER component play during NER is more significant than that of GG-NER components at early developmental stages.

Taken together, these results suggest that the *Arabidopsis* RAD7/16 (the yeast-like GG-NER path) makes a significant contribution to plant NER compared to the CSB/UVSSA and DDB1/2 pathways. These data agree with our plant NER model, in which we hypothesized AtRAD16, AtDDB2, and AtCSB/UVSSA contribute to the GG-NER and NER pathways, respectively.

## Figures and Tables

**Table 1:** Seed abortion rates based on physical appearance.

Genotypes	Wt. Green Seeds	Empty Spots	Small White Dot	Small Brown	Medium Brown	Total
Col	870	0	0	0	0	870
rad16-2	740 (99.6 %)	0	0	3 (0.4%)	0	743
rad16-2 rad23b/+	600 (75.09%)	10 (1.25%)	10 (1.25%)	79 (9.88%)	100 (12.51%)	799
rad23b	780	0	0	0	0	780
Rad23b rad16-2/+	589 (78.22%)	6 (0.79%)	3 (0.39%)	73 (9.69%)	82 (10.88%)	753

## Supplementary Materials

The following supporting information can be downloaded at: https://www.mdpi.com/article/10.3390/genes14081552/s1, Table S1: List of primers used in this study (sequence (5′ to 3′)); Table S2: Percent of identity between yeast and *Arabidopsis* RAD16 protein sequences; Figure S1: Sequence analysis of yeast and *Arabidopsis* RAD16 proteins; Figure S2: Visualization of *Arabidopsis RAD16* and *RAD16b* expression level from AtGenExpress; Figure S3: Visualization of *Arabidopsis RAD16* and *RAD16b* expression level following UV treatment; Figure S4: *Arabidopsis rad16* and *rad16b* single and double null mutants exhibit a UV sensitive phenotype in adults; Figure S5: Adult developmental phenotypes of *Arabidopsis rad16b* and *rad16* single and double mutants; Figure S6: *Arabidopsis* RAD16 overexpression rescues early flowering time and short silique length phenotypes; Figure S7: *Arabidopsis* YFP-RAD16 overexpression rescues the early flowering time and short silique length phenotypes; Figure S8: Yeast two-hybrid screening for *Arabidopsis* RAD16 and RAD7a self-interaction; Figure S9: Yeast two-hybrid screening for *Arabidopsis* RAD16/RAD7b interaction.; Figure S10: Yeast two-hybrid screening for *Arabidopsis* RAD16/RAD4 interaction.; Figure S11: Yeast two-hybrid screening for *Arabidopsis* RAD16/DDB2 interaction.; Figure S12: Yeast two-hybrid screening for *Arabidopsis* RAD16/ELOC interaction; Figure S13: Yeast two-hybrid screening for *Arabidopsis* RAD16/RAD23b interaction.
