# Supplementary material for: Arabidopsis RAD16 Homologues Are Involved in UV Tolerance and Growth
Source: Genes (Basel). 2023 Jul 28;14(8):1552. doi: 10.3390/genes14081552 (PMC10454142; doi:10.3390/genes14081552)
Supplement: Supplementary file 1 [file genes-14-01552-s001.zip › genes-2507409-supplementary.pdf]

Supplementary Materials:

Supplemental Table S1. List of primers used in this study (sequence (5' to 3'))

**Genotyping**				
RAD16	RAD16-2F	TTGTTAGTATGTGGCAAGCC	RAD6-2R	GCACATACCAAAGCCTCAATC
RAD16b	RAD1b-1F	TAGAAACGATGCGGTACCTTG	RAD16b-1R	GTCATGTTGGTTGCTTCTTCG
RAD7a	RAD7a-3R	ATCTATAAGTGGTGCATGCC	RAD7a-1R	AAAAAGAAGCAAAGCAGAGGG
RAD7c	RAD7c-521F	GAAGGCCTTGGCTTATGCT	RAD7c-1R	TTCTTCCATCTCCACGTCTTG
DDB2	DDB2.3F	ACGACGTGTTTTGTCGGTGTGGAAGAA	DDB2-3R	ATAGCAGGAGCTTTACCAGGC
CSB	CSB-1L	TACCGTTTCAACAAAACCAGC	CSB-1R	TCTTTGACGAAACCAGTTTCG
UVSSA	UVSSA-2L	GAGCAAGAAGCCATTGAGATG	UVSSA-2R	CTGTCTCTCTCGTTGAATCCG
RAD23b	RAD23b-30	GTCTTACCTGAAGAATTTGAGGGTTGG	RAD23b-31	CCTGTTTCCGCTACCACATCTTCGACT
SALK	LBb1.3	ATTTTGCCGATTTCGGAAC		
**Semi-quantitative RT-PCR**				
RAD16	RAD16c-1211F	GCTTCTTGCAAATTCGCCCA	RAD16c-2147R	ATGGTCATGCTTCCCACCAG
RAD16b	RAD16Bc-129F	TGTGGGTGTAAGGTCTCGTG	RAD16Bc-346R	TTCACCGCCGACTGAATCATRAD1
ACTIN	ACT- F	CTGGAACAAGACTTCTGGGC	ACT- R	GGTGATGAAGCACAATCCAAG
**qPCR**				
RAD16	RAD16Q5	TGAGACAGGCCGTTGATCAT	RAD16Q6	CAGAGACCACTTCTTGCTCA
EF1A	EF1AQ3	CTGGAGGTTTTGAGGCTGGT	EF1AQ4	GGTGGTGGCATCCATCTTGT
**Construct the recombinant plasmids**				
SfiA N RAD16		GAATTCGGCCGTCAAGGCCAGAAGGAGATATAACCATGGAGCTTCGGTCCCGCAAT		
RAD16 C SfiB		AGTCGACGGCCCATGAGGCCTTATGTTGTAAA		

Supplemental Table S2. Percent of identity between yeast and *Arabidopsis* RAD16 protein sequences

	ScRAD16	SpRHP16	AtRAD16	AtRAD16b
ScRAD16		52%	40%	34%
SpRHP16			40%	35%
AtRAD16				61%

```
                 1                                                50
AtRAD16     (1)  --------------------------------------------------
AtRAD16b    (1)  --------------------------------------------------
ScRAD16     (1)  --------------------------------------------------
SpRHP16     (1)  MGTSCNKINSNSNKGKENMHFVLDDNGDSKGNASNQQVERDDKLDMETTR
                 51                                               100
AtRAD16     (1)  --------------------------------------------------
AtRAD16b    (1)  --------------------------------------------------
ScRAD16     (1)  ----------MQEGGFIRRRRTRSTKKSVNYNELSDDDTAVKNSKTLQLK
SpRHP16    (51)  WNGKEFEEPLSTNKKLIIQSNNTSSQHSTPPLSISDTSTHTG-SSTDNVE
                 101                                              150
AtRAD16     (1)  --------------MELRSRNKAIRPSTEVVDLEEGTGINPDEEPYAISS
AtRAD16b    (1)  --------MAGLRSVHVGSETKVVS-KNECAHVTLWTGTAQGDLGVAMEP
ScRAD16    (41)  GN------SENVNDSQDEEYRDDATLVKSPDDDDKDFIIDLTGSDKERTA
SpRHP16   (100)  ANPNTGFSSARKRSLRSSNLKKKFVPLSSPEESNESEFIDDDESDEVASI
                 151                                              200
AtRAD16    (37)  DDDSIGSEFQGDEEEEEELEE-VVANDDLPNPVPVLAIVNLPRASKKRKK
AtRAD16b   (42)  HSHHKNAILPSSSQDENLKEE-EVPDDDDSVGGEVQGEVNANDYIPNPAA
ScRAD16    (85)  TDENTHAIKNDNDEIIEIKEERDVSDDDEPLTK-----KRKTTARKKKKK
SpRHP16   (150)  IDIKEDETFDSKVEIPEAAPSSSTESDEESIPLSYQSKRRRVSARASSSA
                 201                                              250
AtRAD16    (86)  --PDARKEKVVLLWETWEKEQNSWIDEHMSEDVDLDQHNAVIAETAEPPS
AtRAD16b   (91)  --PANTKRKWQIMKEKVQ----------MTEDDDFDEQNAVIAEAAEQPL
ScRAD16   (130)  -TSTKKKSPKVTPYERNTLRLYEHHPELRNVFTDLKNAPPYVPQRSKQPD
SpRHP16   (200)  SSSSRTQAKSIPSHERTHYRLIRQHPELEHVWEKLEEEAPREVKQIEQPK
                 251                                              300
AtRAD16   (134)  DLIMPLLRYQKEFLAWATKQEQ-SVAGGILADEMGMGKTIQAISLVLARR
AtRAD16b  (129)  DLIIPLLKYQKEFLAWATIQELSAVRGGILADEMGMGKTIQAISLVLARR
ScRAD16   (179)  GMTIKLLPFQLEGLHWLISQEESIYAGGVLADEMGMGKTIQTIALLMNDL
SpRHP16   (250)  ELVLNLLPFQREGVYWLKRQEDSSFGGGILADEMGMGKTIQTIALLLSEP
                 301                                              350
AtRAD16   (183)  EVDRAQFGEAAGCTLVLCPLVAVSQWLNEIARFTSPGSTKVLVYHGAKRA
AtRAD16b  (179)  EVDRAKSREAVGHTLVLVPPVALSQWLDEISRLTSPGSTRVLQYHGPKRD
ScRAD16   (229)  TKSPS---------LVVAPTVALMQWKNEIEQHTK-GQLKIYIYHGASRT
SpRHP16   (300)  RGKPT---------LVVAPVVAIMQWKEEIDTHTN-KALSTYLYYGQARD
                 351                                              400
AtRAD16   (233)  KNIKEFMNYDFVLTTYSTVESEYRRNIMPSKVQCAYCSKSFYPKKLVIHL
AtRAD16b  (229)  KNVQKLMNYDFVLTTSPIVENEYR--------------------------
ScRAD16   (269)  TDIKDLQGYDVVLTTYAVLESVFR--------------------------
SpRHP16   (340)  ISGEELSSYDVVLTSYNVIESVYR--------------------------
                 401                                              450
AtRAD16   (283)  RYFCGPSAVKTAKQSKQKRKKTSDSSSQQGKEADAGEDKKLKKSKKKTKQ
AtRAD16b  (253)  ---------------------------K------------D---------
ScRAD16   (293)  ------------------------------------------------KQ
SpRHP16   (364)  ------------------------------------------------KE
                 451                                              500
AtRAD16   (333)  TVEKDQLGSDDKEKSLLHSVKWNRIILDEAHYIKERRSNTARAVFALEAT
AtRAD16b  (255)  --EG--V---DETMSPLHSIKWNRIIVDEAHDIKNRSSRTAKAVFALEAT
ScRAD16   (295)  NYGFRRKNGLFKQPSVLHNIDFYRVILDEAHNIKDRQSNTARAVNNLKTQ
SpRHP16   (366)  RSGFRRKNGVVKEKSLLHQMEFYRIILDEAHGIKSRTCNTARAVCGLRTT
                 501                                              550
AtRAD16   (383)  YRWALSGTPLQNRVGELYSLIRFLQIRPYSYYFCK--DCDCRILDYVAHQ
AtRAD16b  (298)  YRWALSGTPLQNDVDELYSLVS------YSFLN----FFYSTYASFAFRH
ScRAD16   (345)  KRWCLSGTPLQNRIGEMYSLIRFLNINPFTKYFCTKCDCASKDWKFTDRM
SpRHP16   (416)  RKICLSGTPLQNRIGELFSLLRFLRADPFAYYYCLQCECKSLHWRFSDRS
```

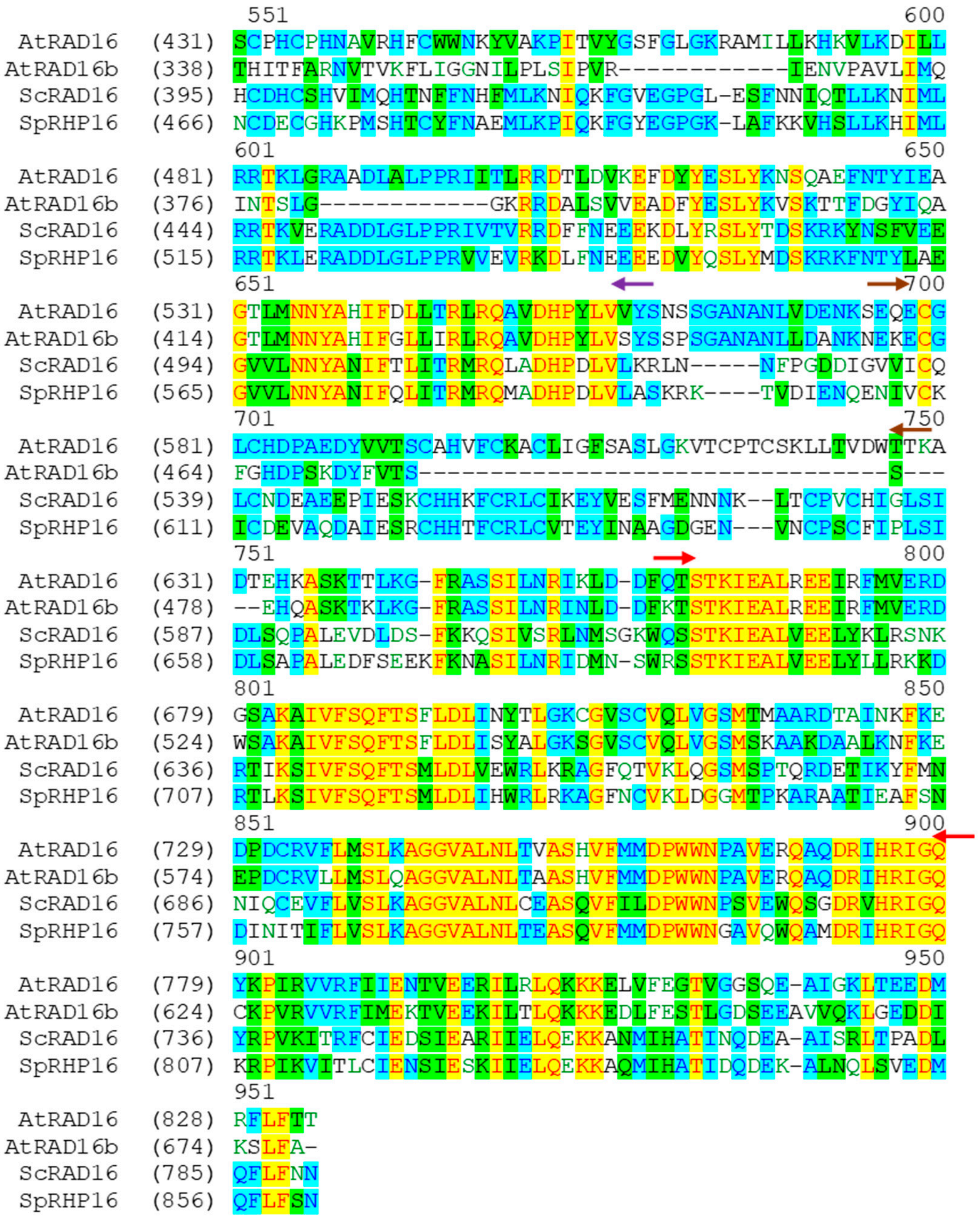

Supplemental Figure S1. Sequence analysis of yeast and *Arabidopsis* RAD16 proteins. RAD16 and RAD16b protein sequences of *Arabidopsis* (NP_172004.1, NP_171767.1), *S. cerevisea* (P31244.1), and S. pombe (P79051.1) aligned using AlignX, helicase ATP binding domain is indicated in black, the start and the stop of snf2-N domain is indicated in purple arrows, zinc finger ring-type in brown, and helicase c-terminal domain in red

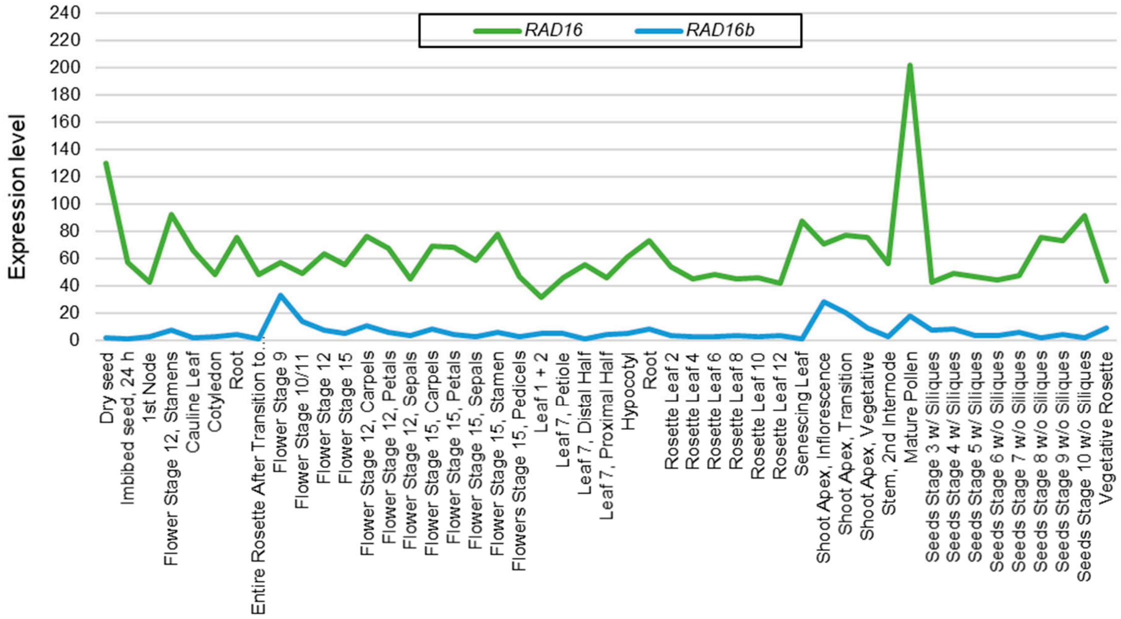

Supplemental Figure S2. Visualization of *Arabidopsis RAD16* and *RAD16b* expression level from AtGenExpress. Expression level of *RAD16* (green) and *RAD16b* (blue) in different tissues during different stages of Arabidopsis thaliana development. Expression values were calculated using The Gene Chip OperatingSoftware (GCOS; TGT=100, Bkg=20). Data were obtained from Schmid et al. (2005) [37].

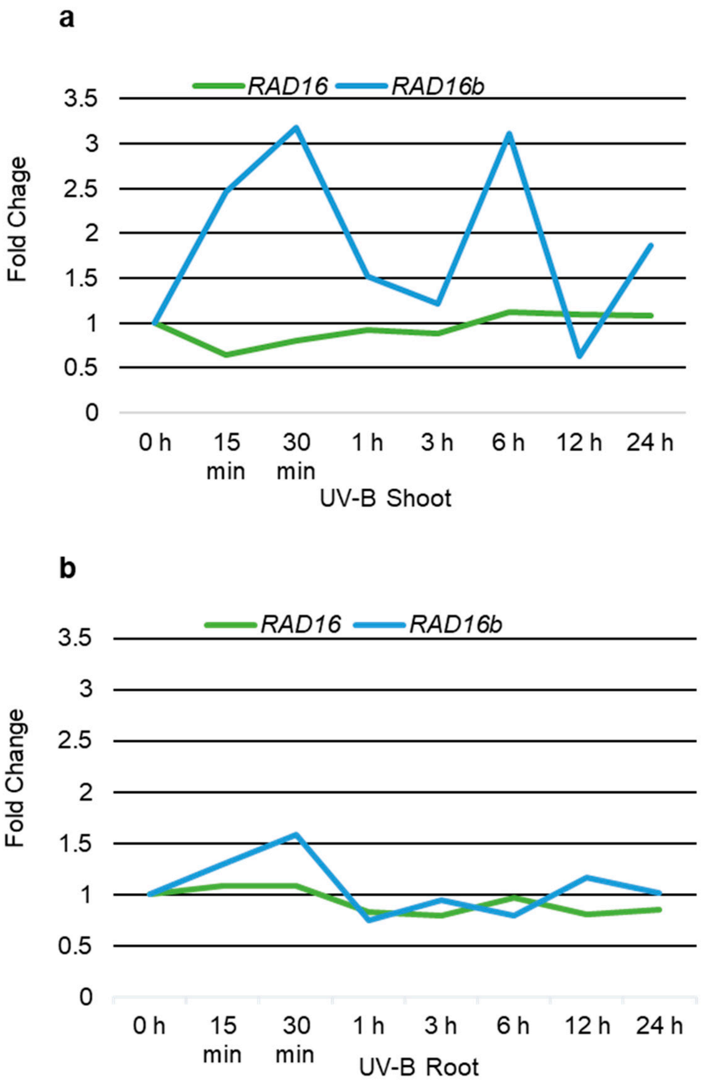

Supplemental Figure S3. Visualization of *Arabidopsis RAD16* and *RAD16b* expression level following UV treatment. Expression level of *RAD16* and *RAD16b* following UV-B treatment in (a) shoot and (b) root tissues relative to untreated control of the same genotype. Data were obtained from Kilian et al. (2007) visualized by Arabidopsis eFP browser [38].

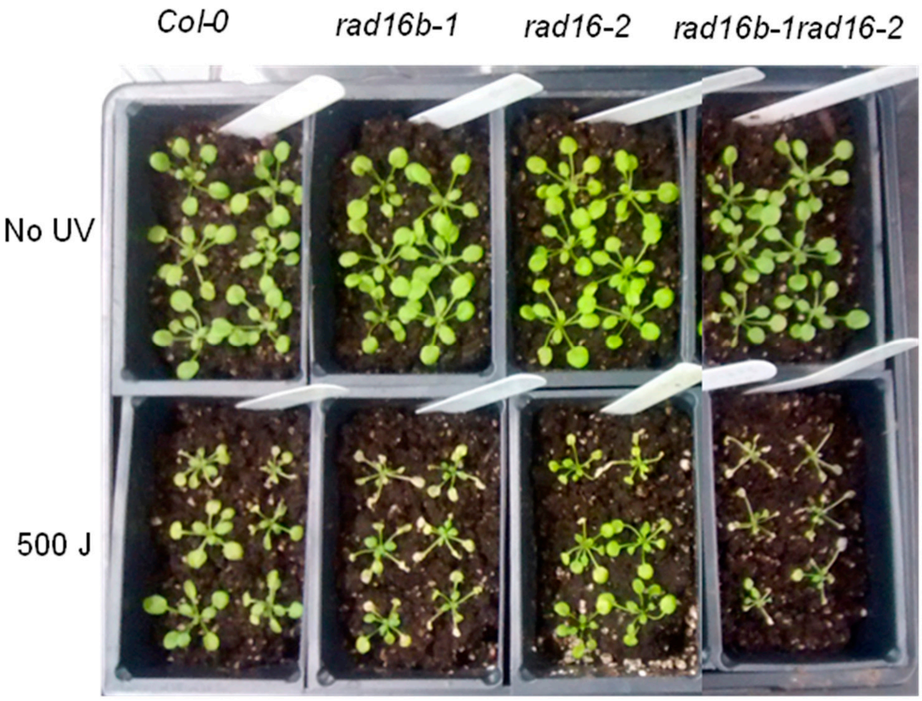

Supplemental Figure S4. *Arabidopsis rad16* and *rad16b* single and double null mutants exhibit a UV sensitive phenotype in adults. 21- day- old plants exposed to 500 J UV-C irradiation, incubated in the dark for three days, followed by four days incubation at (16h light/8h dark).

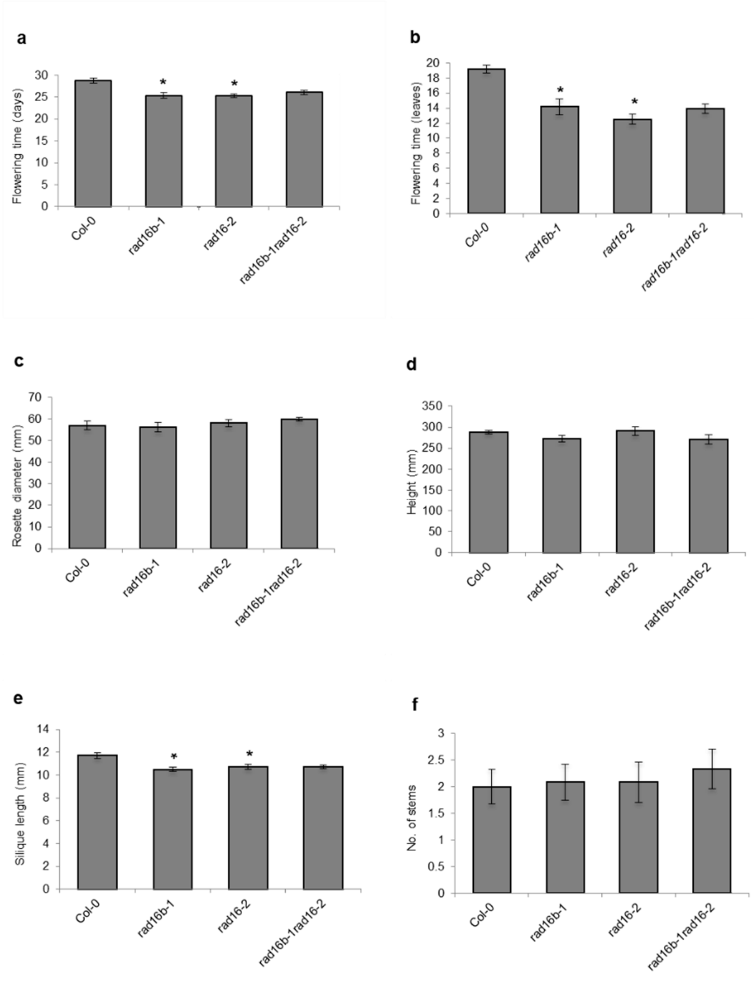

Supplemental Figure S5. Adult developmental phenotypes of *Arabidopsis rad16b* and *rad16* single and double mutants. (a) Flowering time, average number of days and (b) average number of leaves. (c) Average rosette diameter (mm). (d) Average height (mm). (e) Average slique length. (f) Apical dominance (average number of stems). Values are means ± SE (n= 12), * = $p \leq 0.05$ of single mutant vs wild type, and double mutants vs *rad16-2*.

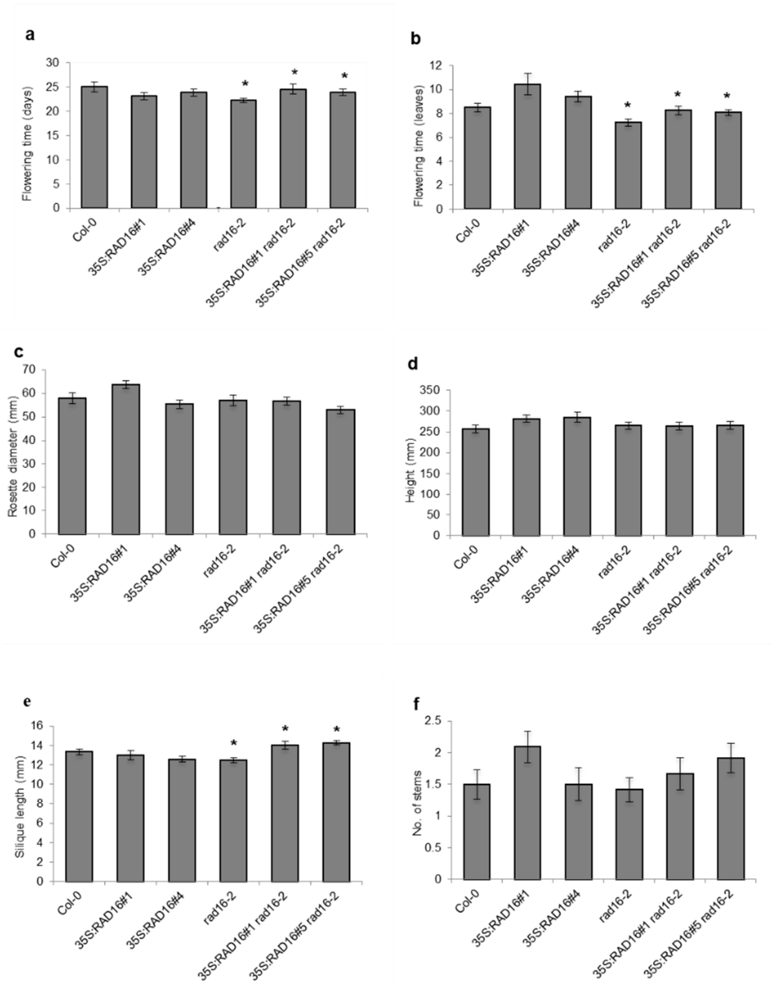

Supplemental Figure S6. *Arabidopsis* RAD16 overexpression rescues early flowering time and short silique length phenotypes. (a) Flowering time, average number of days and (b) average number of leaves. (c) Average rosette diameter (mm). (d) Average height (mm). (e) Average slique length. (f) Apical dominance (average number of stems). Values are means ± SE (n= 12), * = $p \leq 0.05$ of rad16-2 and *35S: RAD16* vs wild type (Col-0) and *35S: RAD16 rad16-2* vs *rad16-2*.

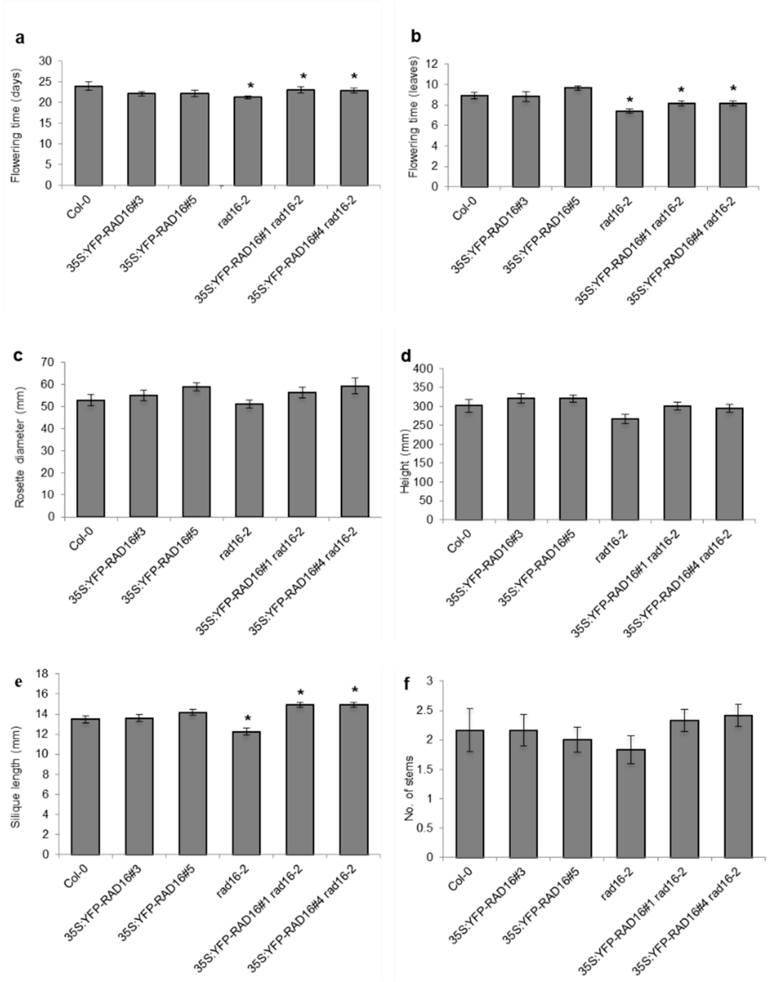

Supplemental Figure S7. *Arabidopsis YFP-RAD16* overexpression rescues the early flowering time and short silique length phenotypes. Flowering time, average number of days (a) and average number of leaves (b). (c) Average rosette diameter (mm). (d) Average height (mm). (e) Average slique length. (f) Apical dominance (average number of stems). Values are means ± SE (n= 12), * = $p \leq 0.05$ of *rad16-2* and *35S: YFP-RAD16* vs wild type (*Col-0*) and *35S:YFP- RAD16 rad16-2* vs *rad16-2*.

a

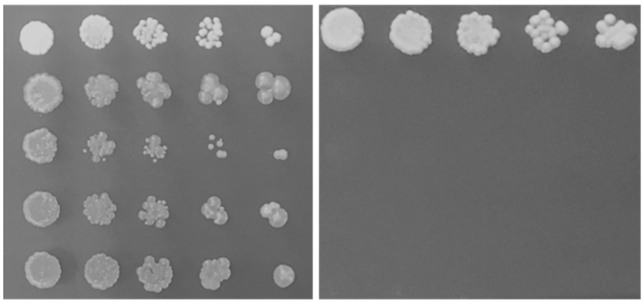

pGBKT7	pGADT7
P53	T
Lam	T
RAD16	-
-	RAD16
RAD16	RAD16

b

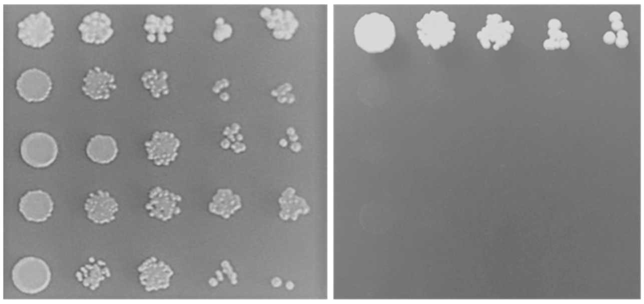

pGBKT7	pGADT7
P53	T
Lam	T
RAD7a	-
-	RAD7a
RAD7a	RAD7a

Supplemental Figure S8. Yeast two-hybrid screening for *Arabidopsis* RAD16 and RAD7a self-interaction. Neither RAD16 nor RAD7a interacted with itself. Each of *RAD16* and *RAD7a* were cloned into pGBKT7 (bait) and pGADT7 (prey) vectors and transformed into haploid yeast cells, then mated. Five-fold dilutions of the mated diploid strains with a starting concentration of 0.2 optical density at 600 nm; RAD16 bait/RAD16 prey (**a**) and RADa bait/RAD7a prey (**b**) were spotted on (-leu -trp) non-selective plates (left) and on selective plates (-leu –trp –ade -his) on the right. P53/T and Lam/T are the positive and negative controls respectively, as P53 interacts with T, whereas Lam does not interact with T.

a

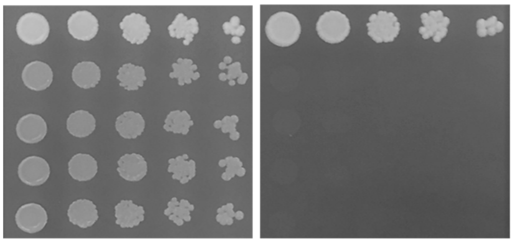

pGBKT7	pGADT7
P53	T
Lam	T
RAD16	-
-	RAD7b
RAD16	RAD7b

b

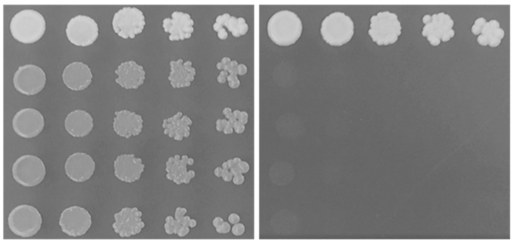

pGBKT7	pGADT7
P53	T
Lam	T
RAD7b	-
-	RAD16
RAD7b	RAD16

Supplemental Figure S9. Yeast two-hybrid screening for *Arabidopsis* RAD16/RAD7b interaction. RAD16 did not interact with RAD7b. Each of *RAD16* and *RAD7b* were cloned into pGBKT7 (bait) and pGADT7 (prey) vectors and transformed into haploid yeast cells, then mated. Five-fold dilutions of the mated diploid strains with a starting concentration of 0.2 optical density at 600 nm; RAD16 bait/RAD7b prey (a) and RADb bait/RAD16 prey (b) were spotted on (-leu -trp) non-selective plates (left) and on selective plates (-leu –trp –ade -his) (right). P53/T and Lam/T are the positive and negative controls respectively, as P53 interacts with T, whereas Lam does not interact with T.

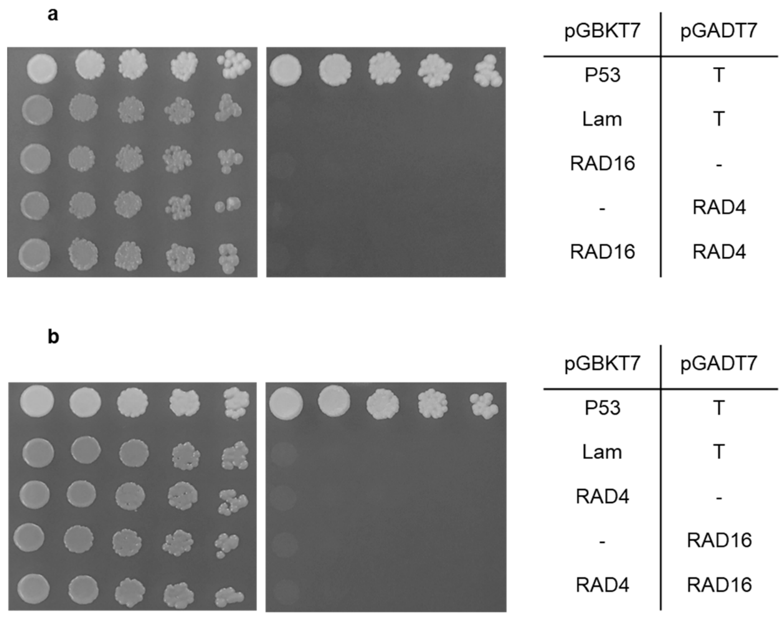

Supplemental Figure S10. Yeast two-hybrid screening for *Arabidopsis* RAD16/RAD4 interaction. RAD16 did not interact with RAD4. Each of *RAD16* and *RAD4* were cloned into pGBKT7 (bait) and pGADT7 (prey) vectors and transformed into haploid yeast cells, then mated. Five-fold dilutions of the mated diploid strains with a starting concentration of 0.2 optical density at 600 nm; RAD16 bait/RAD4 prey (**a**) and RAD4 bait/RAD16 prey (**b**) were spotted on (-leu -trp) non-selective plates (left) and on selective plates (-leu –trp –ade -his) (right). P53/T and Lam/T are the positive and negative controls respectively, as P53 interacts with T, whereas Lam does not interact with T.

a

b

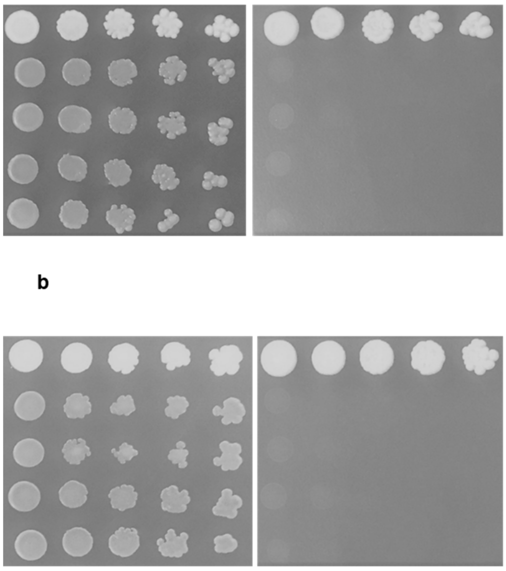

a

pGBKT7	pGADT7
P53	T
Lam	T
RAD16	-
-	DDB2
RAD16	DDB2

b

pGBKT7	pGADT7
P53	T
Lam	T
DDB2	-
-	RAD16
DDB2	RAD16

Supplemental Figure S11. Yeast two-hybrid screening for *Arabidopsis* RAD16/DDB2 interaction. RAD16 did not interact withDDB2. Each of *RAD16* and *DDB2* were cloned into pGBKT7 (bait) and pGADT7 (prey) vectors and transformed into haploid yeast cells, then mated. Five-fold dilutions of of the mated diploid strains with a starting concentration of 0.2 optical density at 600 nm; RAD16 bait/DDB2 prey (a) and DDB2 bait/RAD16 prey (b) were spotted on (-leu -trp) non-selective plates (left) and on selective plates (-leu –trp –ade -his) right. P53/T and Lam/T are the positive and negative controls respectively, as P53 interacts with T, whereas Lam does not interact with T.

a

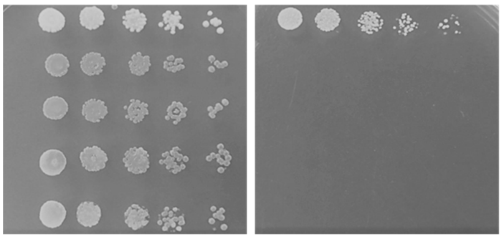

pGBKT7	pGADT7
P53	T
Lam	T
RAD16	-
-	ELOC
RAD16	ELOC

b

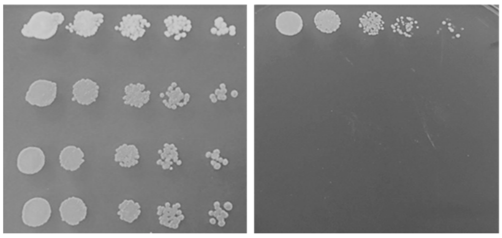

pGBKT7	pGADT7
P53	T
Lam	T
ELOC	-
-	RAD16
ELOC	RAD16

Supplemental Figure S12. Yeast two-hybrid screening for *Arabidopsis* RAD16/ELOC interaction. RAD16 did not interact with ELOC. Each of *RAD16* and *ELOC* were cloned into pGBKT7 (bait) and pGADT7 (prey) vectors and transformed into haploid yeast cells, then mated. Five-fold dilutions of the mated diploid strains with a starting concentration of 0.2 optical density at 600 nm; (a) RAD16 bait/ELOC prey and (b) ELOC bait/RAD16 prey were spotted on (-leu -trp) non-selective plates (left) and on selective plates (-leu –trp –ade -his) (right). P53/T and Lam/T are the positive and negative controls respectively, as P53 interacts with T, whereas Lam does not interact with T.

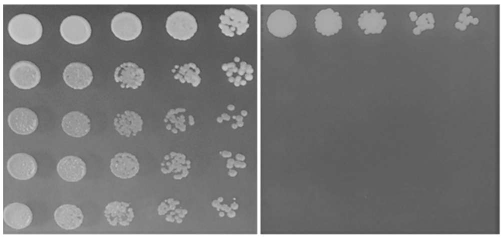

pGBKT7	pGADT7
P53	T
Lam	T
RAD16	-
-	RAD23b
RAD16	RAD23b

Supplemental Figure S13. Yeast two-hybrid screening for *Arabidopsis* RAD16/RAD23b interaction. RAD16 did not interact with RAD23b. Each of RAD16 and RAD23b were cloned into pGBKT7 (bait) and pGADT7 (prey) vectors, respectively, and transformed into haploid yeast cells, then mated. Five-fold dilutions of the mated diploid strain with a starting concentration of 0.2 optical density at 600 nm; RAD16 bait/RAD23b prey were spotted on (-leu -trp) non-selective plates (left) and on selective plates (-leu –trp –ade -his) (right). P53/T and Lam/T are the positive and negative controls respectively, as P53 interacts with T, whereas Lam does not interact with T.
